# Neural correlates of recalled sadness, joy, and fear states: a source reconstruction EEG study

**DOI:** 10.3389/fpsyt.2024.1357770

**Published:** 2024-04-04

**Authors:** Alice Mado Proverbio, Federico Cesati

**Affiliations:** ^1^ Cognitive Electrophysiology Lab, Department of Psychology, University of Milano-Bicocca, Milan, Italy; ^2^ NEURO-MI Milan Center for Neuroscience, Milan, Italy

**Keywords:** social neuroscience, affective neuroscience, EEG/ERPs, emotion, brain-computer interface

## Abstract

**Introduction:**

The capacity to understand the others’ emotional states, particularly if negative (e.g. sadness or fear), underpins the empathic and social brain. Patients who cannot express their emotional states experience social isolation and loneliness, exacerbating distress. We investigated the feasibility of detecting non-invasive scalp-recorded electrophysiological signals that correspond to recalled emotional states of sadness, fear, and joy for potential classification.

**Methods:**

The neural activation patterns of 20 healthy and right-handed participants were studied using an electrophysiological technique. Analyses were focused on the N400 component of Event-related potentials (ERPs) recorded during silent recall of subjective emotional states; *Standardized weighted Low-resolution Electro-magnetic Tomography* (swLORETA) was employed for source reconstruction. The study classified individual patterns of brain activation linked to the recollection of three distinct emotional states into seven regions of interest (ROIs).

**Results:**

Statistical analysis (ANOVA) of the individual magnitude values revealed the existence of a common emotional circuit, as well as distinct brain areas that were specifically active during recalled sad, happy and fearful states. In particular, the right temporal and left superior frontal areas were more active for sadness, the left limbic region for fear, and the right orbitofrontal cortex for happy affective states.

**Discussion:**

In conclusion, this study successfully demonstrated the feasibility of detecting scalp-recorded electrophysiological signals corresponding to internal and subjective affective states. These findings contribute to our understanding of the emotional brain, and have potential applications for future BCI classification and identification of emotional states in LIS patients who may be unable to express their emotions, thus helping to alleviate social isolation and sense of loneliness.

## Introduction

The ability to communicate one’s emotional state is at the basis of social behavior (1). Asking for help when we are scared, comforting when we are sad and sharing our joy when we are happy are psychological needs dictated by our being social animals (2). Despite the importance of these innate needs, there have been few neuroscientific studies of the neural signals associated with inner motivational states in people who are unable to communicate verbally. For example, in Brain Computer Interface (BCI) studies, the recording and classification of electrical potentials is used to infer the mental content of patients with locked-in syndrome (LIS, 3). Patients who are conscious and can generate motor commands or readiness potentials (4, 5), or can make voluntary decisions by generating P300 components (6), can communicate by controlling cursors, robots, prostheses, speller systems (7), or objects with their volitional signals. However, patients in a vegetative state, also known as unresponsive wakefulness syndrome (UWS) (8), or in a minimally conscious state (9), are cut off from these systems (10). Neuroscientists are researching methods to detect their motivational or emotional states from their brain activity (11). This category includes studies that observe brain activation to infer innate mental content. Owen et al. (12) was the first study to utilize functional magnetic resonance imaging (fMRI) in evaluating the capacity of patients with disorders of consciousness to understand and comply with instructions. They conducted the study on a patient diagnosed as UWS, who was instructed to imagine playing tennis, navigating through her house, and rest without particular thought in blocks of 30 seconds while in the MRI scanner. The design of the study ensured that the patient’s responses were not simply a result of passive processing of verbal instructions, and that they were absent when instructed not to perform a task. The activation of specific brain regions, such as the supplementary motor area during tennis imagery and the parahippocampal gyrus during navigation imagery, allowed for measurement of the patient’s ability to follow specific commands, similar to what is observed in healthy individuals. In a recent ERP study, Proverbio et al. (13) examined the psychophysiological markers of imagery processes. Participants were shown visual and auditory stimuli representing different semantic categories and were then asked to activate a mental image corresponding to the category. The authors were able to identify unique electrophysiological markers of different imagined stimulus classes (e.g., infants, human faces, animals, music, speech, affective vocalizations and sensory modality (visual *vs*. auditory), without sensory stimulation. These ERP signals were then classified by machine learning algorithms (MIRACLE’s classification, 14) surpassing the 70% threshold for effective communication, with accuracy rates of 96.37% and 83.11% in k-fold cross-validation and hold-out validation, respectively. Affective computing is a branch of AI that deals with emotions. It includes automatic emotion recognition, which is currently advancing due to the availability of affordable devices for recording brain signals (15–17). Two studies measured alpha and beta EEG frequencies during the induction of emotions with images, audio or clips thought to induce specific affective states, and performed signal classifications. In particular, Choppin (18) achieved a 64% success rate by analyzing EEG signals and using neural networks to classify them into six emotions based on emotional valence and arousal. In another study, Takahashi (19) used statistical feature vectors previously used for emotion recognition from physiological signals. They conducted a user-independent emotion recognition study using physiological and EEG signals. From the EEG signals alone, a success rate of approximately 41.68% was achieved, and when the physiological and EEG signals were combined, the success rate was 41.72%. These results were obtained from data collected from 12 subjects and involved the discrimination of five different emotions: happiness, anger, sadness, fear and relaxation. With a different approach Proverbio and Pischedda (20), recorded brain signals linked to imagined motivational and emotional states by recording ERPs synchronized with luminance changes preceded by pictograms and found that anterior N400 and centroparietal late positive potential were modulated by subjective recalled states of sadness, fear and joy. The aim of the present investigation was to reconstruct the individual patterns of brain activity recorded during those emotional states, in order to develop methods for identifying mental states based on patterns of brain activation, as demonstrated in Owen et al.’s (12) study. We focused on fear, sadness, and joy as emotions that may be most effective in promoting emotional communication to alleviate the patient’s sense of social isolation. This was done to ensure that the protocol was effective in promoting emotional communication and alleviating the patient’s sense of social isolation. The experimental protocol was refined by modelling the conditions of motor paralysis, absence of verbal communication, and eye movement in healthy participants.

### Emotional imagery

Lang’s bio-informational theory (21) suggests that an emotionally arousing stimulus can activate the same neural networks as if the stimulus was experienced in real life. Imagery is powerful in evoking strong emotional responses and has been linked to various clinical conditions and therapies. For example, in the case of Post-Traumatic Stress Disorder (PTSD), emotional imagery can trigger strong emotions and flashbacks of traumatic events. Additionally, in the context of dependencies, imagining the use of a drug can cause desires or cravings for the substance (22). Indeed, due to its ability to evoke emotion-related images, imagery has been incorporated into psychological treatments and therapeutic approaches. This integration assists patients in modifying the content of emotion-inducing imagery, especially in cases of PTSD and social phobia (23–25). Overlap exists between the processes involved in mental imagery and perception, which can lead individuals to respond “as if” they are experiencing real emotion-arousing events. Research has shown that emotional content, such as facial expressions, activates specific brain areas (26), resembling the neural activation observed during actual perception (27). Again, Marmolejo-Ramos et al. (28) and Suess and Abdel Rahman (29) have shown that imagination of emotional stimuli involves brain activations similar to those present during perception, suggesting a connection between perceptual and emotional processes.

### The emotional neural network

Emotional states can be studied during simulation and mental recall, as in the present experimental paradigm. Indeed, studies have shown that mental images of emotional states can be generated by recalling memories for emotional episodes in the past, by reliving the feelings associated with past events stored in autobiographical memory or by generating new feelings based on the perceptual content of the constructed image itself (30). A recent neuroimaging study (31) found that distinct neural foundations underlie various emotions, characterized by unique activation patterns across extensive cortical and subcortical networks (32). The differentiated engagement of these neural circuits gives rise to distinct neural activity patterns, which in turn correspond to the subjective feelings associated with each emotion. This suggests that the brain represents a multitude of emotions in a distinguishable manner, even though there is some overlap in the brain regions involved (31). Each emotion appears to modulate different functional systems within the brain, resulting in unique emotional states (33). For example, while some emotions may share certain sensory representations, their underlying internal representations may differ. This leads to the formation of distinct emotional states based on the general configuration of the central and peripheral nervous systems. At this regard, Saarimaki et al. (31) conducted an exploratory analysis that used hierarchical clustering to identify four clusters within the neural data representing emotion-specific patterns. These clusters aligned with categories of emotions, including positive emotions (e.g., pride, longing, happiness, gratitude, and love), negative basic emotions (such as disgust, sadness, fear, and shame), negative social emotions (like anger, guilt, contempt, and despair), and the emotion of surprise. When comparing the subjective experience of emotions with the similarity of their neural patterns, a direct link emerged. Emotions with more similar neural signatures tended to be subjectively experienced as more alike.

Furthermore, specific brain regions consistently exhibited activation patterns during various emotional experiences. Midline brain regions, including the anterior cingulate cortex (ACC), posterior cingulate cortex (PCC), and precuneus, were active during most emotions (34–38). These regions are believed to encode emotional valence, engage in self-relevant introspection, and integrate information concerning internal, mental, and bodily states (35). Subcortical regions, such as the amygdala and thalamus, displayed distinct activation patterns that varied across emotion clusters (39–42). These regions are associated with processing emotional significance and arousal and exhibit unique activation patterns for both basic and non-basic emotions (32). Other brain regions, including the premotor cortex, cerebellum, basal ganglia and posterior insula, were active during emotions associated with avoidance (e.g., fear, disgust, sadness, shame, surprise) (33). Moreover, the anterior prefrontal cortex demonstrated activation primarily during positive emotions (e.g., happiness, love, pride, gratitude, and longing), in line with prior research linking this region to positive emotional states (43–45). Notably, activation of the orbitofrontal cortex is associated with processing rewards, joy and gratification (46).

### Neural bases of fear

Fear emotion has been crucial to the survival and adaptation of human species throughout evolutionary history (47). This emotion triggers an intricate interplay of cognitive, physiological, and behavioral processes in response to potential threats (48). Animal studies (49) have shown the existence of a circuit for the regulation of fear and anxiety including the amygdala, periaqueductal grey matter, hippocampus, and prefrontal cortex, while studies in humans have highlighted the role of limbic area and amygdala nuclei (50–53). Peñate et al. (54) have especially highlighted the key role of limbic areas in fear sensation. In a meta-review they examined functional magnetic resonance imaging (fMRI) studies of individuals with specific animal phobia compared to healthy controls, and found a high overall effect size for both limbic and frontal sites. Data analyses showed greater brain activity in the left amygdala and insular cortex in phobic individuals. They also observed an activation of the fusiform gyrus, the dorsolateral prefrontal cortex left, and the left cingulate cortex. Again, Rosenbaum et al. (55) investigated the neural dynamics of spider phobia with combined functional near-infrared spectroscopy (fNIRS) and electroencephalography (EEG) and found an increased activation of superior parietal, limbic and prefrontal regions during processing of fearful material (similarly to 56–58). Two independent reviews, which comprehensively analyzed more than 70 papers on phobia consistently pinned down hyperactivation of the fear network of the amygdala, ACC, and insula to phobia-relevant stimuli in phobic patients (59, 60).

### Neural bases of sadness

A recent meta-analysis by Wager and coauthors (61) used Machine Learning analysis to compare brain activity patterns across different emotions. The study showed that the cingulate, insular, and somatosensory areas, which convey information about internal states and visceral sensations, were particularly active during sadness. Additionally, regions in the default mode network, such as the ventromedial prefrontal cortex and hippocampus, supporting self-related sociocognitive processes (62), are also engaged during sadness. This suggests that sadness might involve a heightened internal focus. To gain deeper insights into the neural processes underlying sadness, it is valuable to refer to a study that compared individuals with depression to a control group using fMRI (63). In this study, researchers found significant differences in activation levels between depressed individuals and the control group in various brain regions, including the right frontal cortex, right and left temporal cortex, and right occipital cortex. Significantly, hyper-activity in the frontal lobe (rumination) has been associated with key characteristics of individuals with depression, encompassing functional irregularities, emotional regulation, and cognitive control (64, 65). Frontal lobe dysfunction in addition to temporal lobe dysfunction may be an important risk factor for the development of depression (66), and several studies suggest that right temporal lobe resections are associated with a greater risk of postoperative depression (67), while other studies have reported increased physiological activity in the right hemisphere of the brain in individuals with depression (68). In a more recent study conducted by Proverbio and colleagues (69), the right middle temporal gyrus exhibited significant activation when participants were exposed to stimuli producing negative affect, negative vocalizations, and sad music with lyrics. Additionally, the right superior temporal gyrus plays a key role in perceiving negative facial expressions (70, 71). The study’s authors concluded that right middle temporal area might play a pivotal role in processing social negative stimuli and in the resulting negative mood.

### Neural bases of joy

According to the meta-analysis by Tanzer and Weyandt (72), including 64 neuroimaging studies, joyful sensations would be associated with enhanced activation in several brain regions including the basal ganglia (18% of regions), cingulate cortex (13% of regions), frontal gyrus (9% of regions), insula (7% of regions), amygdala (6% of regions), thalamus (6% of regions), orbitofrontal cortex (4% of regions), and fusiform gyrus (4% of regions). The notable activation observed in the basal ganglia hints at a strong correlation between happiness and movement, as also advanced by Csikszentmihalyi (73). The meta-analysis found that structures in the frontal lobe, which are associated with executive functions like decision-making and cognitive focus (74), were active during happy moods. The orbitofrontal cortex, which processes the value of sensory information, also showed significant activity, thus suggesting that pleasure-associated happiness has a visceral dimension. Given that the orbitofrontal (OBF) cortex, thalamus, and hypothalamus are implicated in sensory information processing or integration (75), their activation may reflect the incorporation of sensory input into the happiness experience. Lastly, the activation of the fusiform gyrus, known for recognizing human faces (76), might be connected to the imaginary activations of people and face images. This could be interpreted as reflecting happiness as a social experience, perhaps associated with interactions with friends.

Based on the literature presented above we expected:

1. To find some areas of activation that were common to the three recalled emotional states, and some that were specific to it (e.g., the right temporal cortex for sadness, the limbic system for fear and OBF reward-related areas for joy). Common areas of activation have been described across all forms of imagery, such as the frontal and parietal regions (77, 78). These areas support short-term memory processes that are essential for the storage and manipulation of information (79, 80), while the occipital area facilitates perceptual experience of imagery (e.g. 81, 82).
2. We also expected that areas supporting recalled emotional states to be part of the circuitry supporting actually felt emotions, for example the left limbic system and amygdala for fearful state (32, 55–58), the orbito-frontal cortex for the joyful state (e.g., 45, 46, 72), and the right temporal lobe for the sadness state (63, 70, 71, 83).

Statistical analyses (more precisely, repeated measure analyses of variance, Wilcoxon signed-rank test and the nonparametric Sign tests) and were performed on the magnitude of source reconstructed electro-magnetic dipoles recorded in a group of 20 participants during recall and imagination of emotional states, to identify and validate reliable markers of emotion-specific brain activity in people absolutely motionless in body and gaze, to simulate Locked-In-Syndrome patients.

## Methods

### Participants

Thirty-one participants (14 males, 17 females), aged about 23 years (SE = 2.73) participated to EEG recordings. 11 participants were excluded for excessive EEG artifacts (in details: 3 participants were discarded for excessive EOG and or VEOG artifacts (threshold = >30% of trials); 3 participants were discarded for excessive alpha noise over posterior leads, 5 participants were discarded for technical problems, such as poor electrode contact, high electrode impedances, and EEG artifacts affecting multiple leads.

The final sample comprised twenty participants, 8 males and 12 females. Their ages ranged from 18 to 35 years (M = 23.20 years, SD = 1.7) and their average education level was 16.8 years of schooling (SD = 1.58). The selection criteria for participation included possessing normal or corrected vision, no existing or prior neurological or psychiatric disorders, and no consumption of any psychotropic drugs or substances that could affect brain activity. Participants were recruited primarily among students of local University through the SONA System website. Each participant received 0.6 University Training Credits (CFU) for their participation. All participants were right-handed, with an average dominance score of 0.84 (SD = 0.17) as assessed through the Edinburgh Inventory. All participants provided written informed consent. The experiment was conducted in accordance with international ethical standards (Helsinki declaration) and was approved by the Research Assessment Committee of the Department of Psychology (CRIP) for minimal risk projects, under the aegis of the Ethical committee of University of Milano-Bicocca (protocol no: RM-2020-242). G*Power analysis (84) was performed to estimate the required sample size, considering the statistical treatment (repeated measures ANOVA), the number of stimulus repetitions (30), and the smallest epsilon value obtained in the ANOVA analysis (0.74). A minimum sample size of 16 Ss (for α = 0.01) was recommended. This indicates a good reliability of a sample size of N=20.

### Stimuli and material

**Figure 1:**
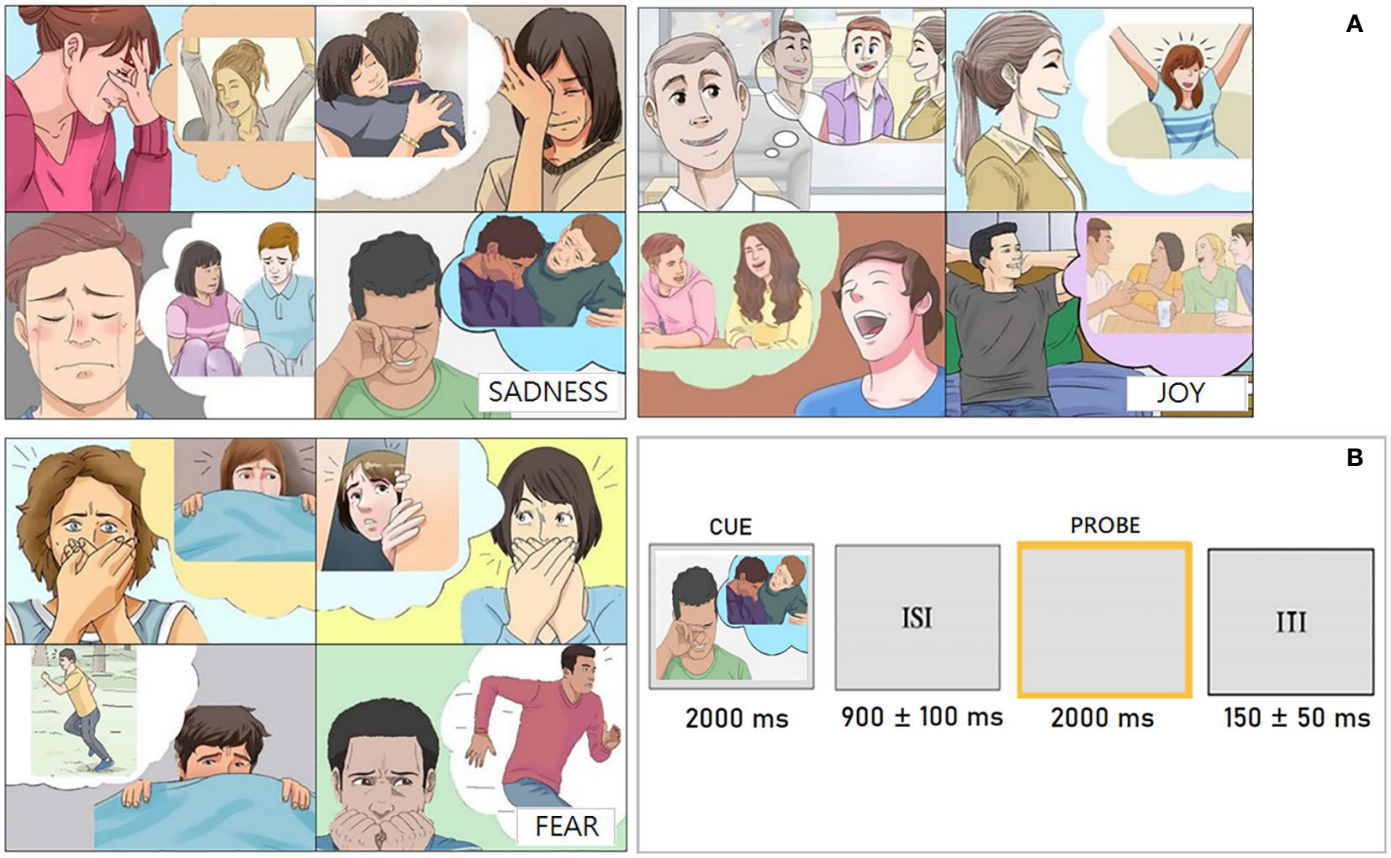
**(A)** Examples of pictograms used to stimulate the recall of affective states belonging to the three types of emotions (sadness, fear and joy) taken from: Proverbio and Pischedda (2023b) (85). **(B)** Outline and timing of the experimental paradigm showing pictogram presentation duration, inter-stimulus interval, probe duration and inter-trial interval.

The stimuli used in this study were sourced from a previously validated Pictionary (85). These stimuli consisted of colored vignettes (**Figure 1A**) depicting male and female individuals who appeared to be young adults. Their facial expressions, contextual cues, and use of pros indicated their emotional state, which fell into one of three categories: sadness, joy, or fear. After the EEG recording, a questionnaire was administered to measure the ease/difficulty with which participants were able to recall various emotional states when prompted by the pictograms. In detail, they were asked to rate the imageability of the situations depicted by pictograms. The emotional contexts depicted in the study received an average rating of 2.61 (SD = 0.40) on a scale of clarity and unambiguity ranging from 0 to 3 (where 0 represents ‘not much’ and 3 represents ‘very much’). This indicates the reliable methodology of the research. The participants were presented with sets of 36 stimuli in a random order. Pictograms were used to visually induce specific emotional states to be recalled. Each stimulus lasted for 2000 ms and was followed by an ISI, which consisted of a blank, illuminated screen lasting between 900 ± 100 ms. The ISI was intended to eliminate any after-images on the retina resulting from the prior stimulation. A bright yellow frame was presented as a visual prompt for imagery. The frame was located in the corner of the screen against a grey background and lasted 2000 ms (**Figure 1B**). The Inter Trial Interval (ITI) was 150 ± 50 ms. Each stimulus was repeated 6 times in different runs for averaging purposes. Participants were given written instructions on how to recreate the emotional state associated with the previously viewed image. They were also required to maintain focus on a specific point during the recording and to evoke a subjective feeling based on their own sensations within a maximum of 5 seconds. It was required that they keep their gaze fixed on the center of the screen. Prior to the EEG recording, participants attended a short training session, which included two 15-stimulus runs. The session aided the participants in comprehending the task requirements.

### EEG recordings

The EEG brain activity was recorded from 128 scalp sites mounted on ECI electro-caps, according to the International 10-5 system. To record horizontal and vertical eye movements and blinks, two electrodes were positioned at the left and right ocular canthi (hEOG), and two above the eyes (vEOG). Reference electrodes were placed behind each ear on the mastoid bone (average mastoid reference), and a ground electrode was positioned at Fz site; for source reconstruction purposes EEG was re-referenced to the average reference.

The impedance of the electrodes was kept below 5 KΩ. The sampling frequency was 512 Hz. The EEG and EOG signals were recorded through the *Cognitrace* program (ANT Software, Enschede, The Netherlands) and amplified with a band-pass filter (0.16-70 Hz). Artifacts with amplitudes greater than ±50 μV were removed before the averaging process. EEG epochs, synchronized with the stimulus presentation (yellow frame acting as probe), were processed using the EEProbe program and started 100 ms before the stimulus presentation. The ERP components were extracted from 100 ms before the stimulus presentation to 1200 ms after the probe presentation. After the averaging process, the ERP components were filtered with a band-pass filter (0.16-15 Hz).

The N400 mean area amplitude values were measured within the 400-600 ms time window, where N400 reached its maximum amplitude (i.e., at anterior frontal and fronto-central sites: FP1, PF2, AF3, AF4, FFC3h, FFC4h, FC1, FC2). The component resembled the fronto/polar N400 previously discussed in literature on imagery-related components (86).

The ANOVA performed on N400 amplitude revealed an effect of “emotion” factor (F (2,38) = 6.65, p <.05). Tukey’s *post-hoc* test revealed that the N400 amplitude was much larger during happiness (M = -1.80 µV, SD = 0.32) than fear imagery states (M = -0.22 µV, SD = 0.44 (20). This time range was selected for source reconstruction in that it proved to be sensitive to the emotional state category.

### Source reconstruction

To identify the cortical sources of the N400 component in response to recalled emotional states of ‘sadness’, ‘fear’, and ‘joy’, three swLORETA models were conducted per participant corresponding to each motivational state, for a total of 60 swLORETAs. *Low-Resolution Electromagnetic Tomography* (LORETA) is a powerful source reconstruction technique used in Brain-Computer Interfaces (BCIs) to localize neural activity with high spatial resolution (87, 88). Utilizing Electroencephalography (EEG) data and a realistic head model with a distributed source model, LORETA avoids the need for restrictive assumptions and efficiently localizes neural sources (89). However, its spatial resolution can be limited in the presence of noise or when multiple dipoles are active simultaneously (87, 88, 90). To address this limitation, Palmero-Soler and colleagues (91) proposed an improved version called SwLORETA, which incorporates a *Singular Value Decomposition* (SVD) based lead field weighting. Additionally, synchronization tomography and coherence tomography based on SwLORETA were introduced to analyze phase synchronization and standard linear coherence, applied to current source density (91).

In comparing LORETA and SwLORETA, recent research by Palmero-Soler et al. (91) demonstrated the superiority of SwLORETA in several aspects: Localization Error: The distance between the maximum of the current distribution and the position of the simulated dipole, referred to as localization error, decreases as the eccentricity increases. SwLORETA shows better performance for all eccentricity and Signal-to-Noise Ratio (SNR) values compared to sLoreta. Activation Volume: Activation volume is the number of voxels with strength above 60% of the maximum Current Source Density (CSD) distribution. SwLORETA focuses the reconstructed CSD around the position of the true dipole, resulting in a smaller activation volume in simulated conditions. Activation Probability: This index is calculated by counting the fractions of times the simulated dipole position is active with a value greater than 60% of the maximum CSD distribution. SwLORETA consistently outperforms LORETA, with the activation probability index being almost always maximal. Overall, the improvements introduced in swLORETA demonstrate its superiority over LORETA in accurately localizing neural sources and enhancing the performance of BCI applications. In conclusion, swLORETA represents a valuable advancement in source reconstruction techniques for BCI applications, offering enhanced spatial resolution and localization performance compared to sLORETA.

For each individual and condition, active dipoles were identified and subsequently categorized based on their Talairach coordinates, Hemisphere, Cerebral area and Brodmann Area (BA). Furthermore they were grouped into seven distinct Regions of Interest (ROIs), as depicted in **Table 1**, following the ROI clustering procedure used to perform statistical analyses on individual LORETA solutions by other authors (92–95).

**Table 1:** ROI clusters used to categorize and quantify individual patterns of activation.

ROI	GYRUS	BRODMANN AREA
OCC Occipital	CUNEUS INFERIOR OCCIPITAL GYRUS LINGUAL GYRUS MIDDLE OCCIPITAL GYRUS SUPERIOR OCCIPITAL GYRUS	17-18-19
TEMP Temporal	INFERIOR TEMPORAL GYRUS MIDDLE TEMPORAL GYRUS SUPERIOR TEMPORAL GYRUS	19-20-21-22-38-39-42
LIMBIC	ANTERIOR CINGULATE CINGULATE GYRUS PPA UNCUS	20-23-24-28-31-34-35-36-38
OBF Orbitofrontal	SUPERIOR FRONTAL GYRUS MIDDLE FRONTAL GYRUS INFERIOR FRONTAL GYRUS SUBCALLOSAL GYRUS RECTAL GYRUS	10-11-44-45-47
FG Fusiform Gyrus	FUSIFORM GYRUS	19-20
DLPF Dorsolateral Prefrontal Cortex	MIDDLE FRONTAL GYRUS MEDIAL FRONTAL GYRUS PRECENTRAL GYRUS SUPERIOR FRONTAL GYRUS	6-8-9-46
AIP	INFERIOR PARIETAL LOBULE PRECUNEUS POSTCENTRAL GYRUS SUPERIOR PARIETAL LOBULE SUPRAMARGINAL GYRUS	7-19-39-40

Abbreviations and extended description of brain areas are reported in the ROI column.

**Figure 2:**
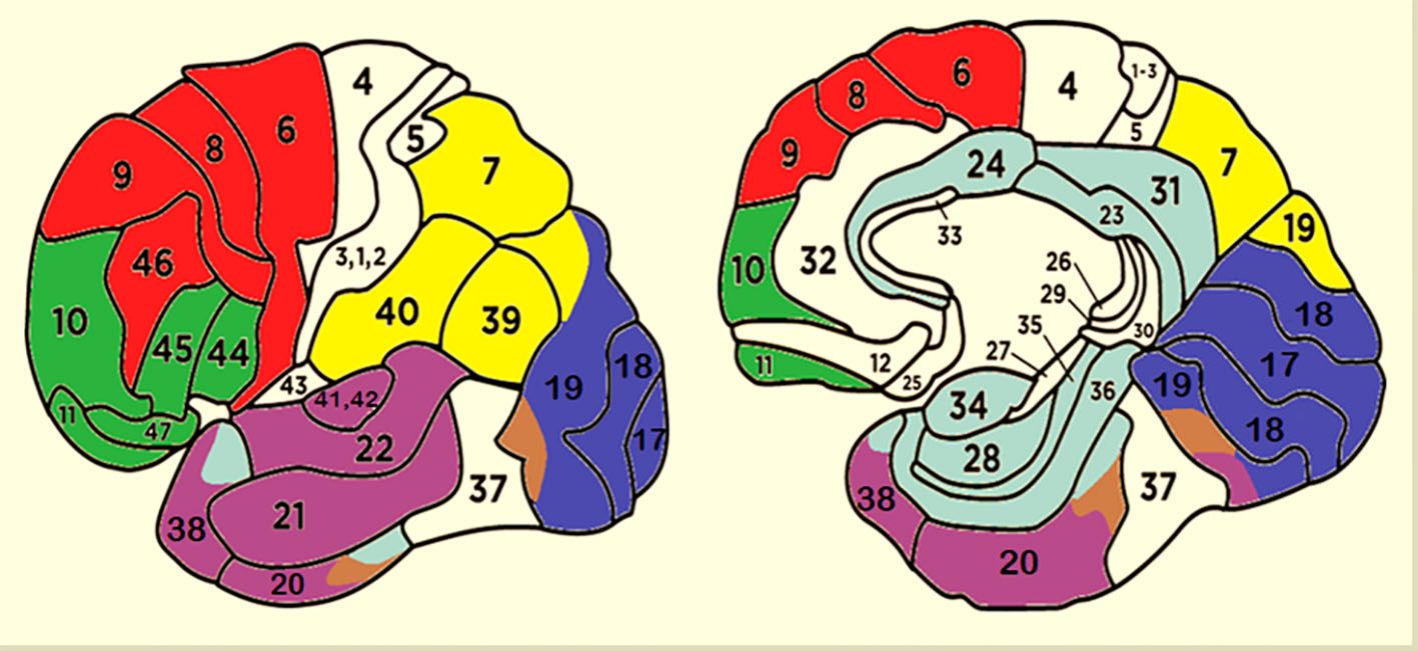
Cluster of areas corresponding to different ROIs. Red, DPLF; Green, OBF; Violet, TEMP; Brown, FG; Blue, OCC; Yellow, AIP; Light Blue, LIMBIC.

Two cortical maps showing the clustering criteria used to generate the different ROIs are provided in **Figure 2**. Only the most active dipole for each ROI was selected. If a participant had no active dipoles in a specific ROI, a value of 0.5 (nA) was assigned for statistical purposes.

Before proceeding with further data analysis, one subject (9AF) was excluded from the study due to the exceedingly noisy EEG signals and excessive EEG artifacts. Additionally, the ROI labelled as AIP (anterior intraparietal area) and DLPF (dorsolateral prefrontal) were removed from the comparison, although being involved in emotional imagery and in the default mode network, as they consistently exhibited some activation level in almost every participant across all three conditions, thereby being poorly distinctive of the specific emotional state. To analyze the neural sources found active in association with the three emotional states, a three-way repeated measures ANOVA was performed on individual activations. Factors were: Emotional state (Sadness, Fear, Joy), ROI: Occipital (OCC), Orbitofrontal (OBF), Temporal (TEMP), Fusiform Gyrus (FG), and LIMBIC; cerebral hemisphere (right and left). Fisher’s LSD and Tukey *post hoc* comparisons were performed to test differences across means. Finally, the distribution of source magnitudes in relevant brain areas was also evaluated using the Wilcoxon signed-rank test and the nonparametric Sign tests. Where appropriate, the Greenhouse-Geisser epsilon correction was applied to control for possible violation of the sphericity assumption. Corrected p-values are reported for epsilon values less than 1.

## Results

**Figure 3:**
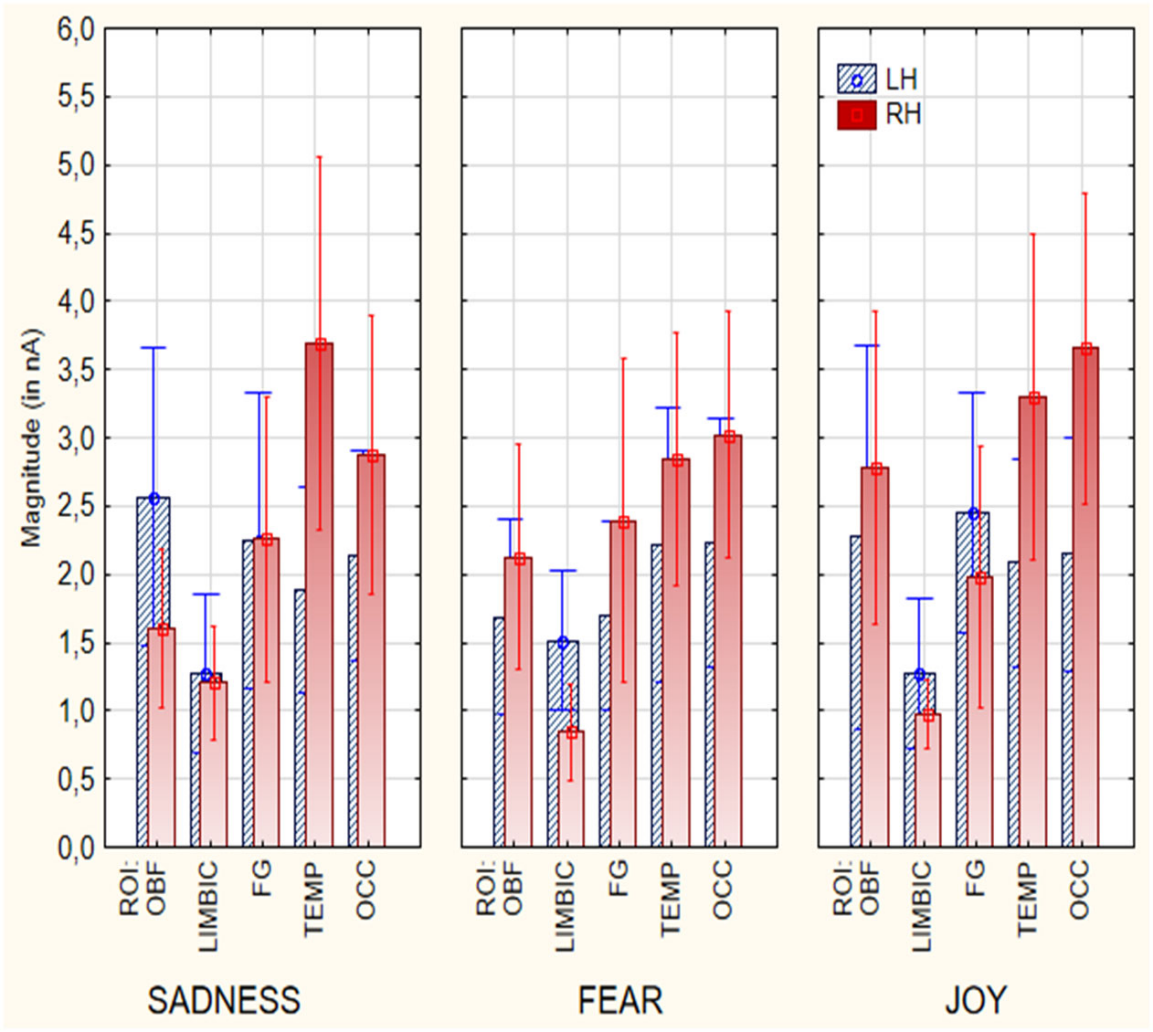
Mean power of electromagnetic sources recorded in different ROIs and cerebral hemispheres as a function of emotional state (in nA).

The results from the ANOVA analysis carried out on the magnitude values of active electromagnetic dipoles (according to SwLORETA) showed the significant effect of Hemisphere [F(1, 18) = 6.27, p < 0.05], with a stronger neural activity over the right hemisphere (M = 2.39 nA, SE = 0.25) than left hemisphere (M = 1.99 nA, SE = 0.18), regardless of emotional state, as visible in **Figure 3**. Furthermore, the results indicated a significant effect of ROI factor [F(4, 72) = 9.17, p < 0.000; ε = 0.82, corr. p value = 0.00003]. *Post-hoc* comparisons revealed that the Orbitofrontal (M = 2.16 nA, SE = 0.33), Fusiform (M = 2.15 nA, SE = 0.31), Temporal (M = 2.70 nA, SE = 0.29), and Occipital ROIs (M = 2.74 nA, SE = 0.27) sent stronger signals than the Limbic area (M = 1.21 nA, SE = 0.10), possibly because of the shorter distance from scalp. Also significant was the interaction of ROI x Hemisphere [F (4, 72) = 3.49, p < 0.05; ε =1]. *Post-hoc* tests indicated that, regardless of emotional states the right Temporal ROI (M = 3.40 nA, SE = 0.47) was the most active than other ROIs. Furthermore, the temporal (left M= 1.99 nA, SE= 0.49; right M= 3.40 nA, SE= 0.47) and occipital (left M= 2.24 nA, SE= 0.48, right M= 3.22 nA, SE= 0. 67) ROIs were more active over the right than the left hemisphere, whereas the limbic ROI was more active over the left (M = 1.40 nA, SE = 0.17) than the right hemisphere (M = 1.02 nA, SE = 0.11). Further significance of the emotional state x ROI x hemisphere interaction [F(8, 14) = 2.16, p < 0.05; ε = 0.74, corr. p value = 0.037], and relative *post-hoc* comparisons, showed that the most active area during the imagined “sadness” condition was the right temporal ROI (M = 3.78 nA, SE = 0.68), compared to all other ROIs, and the left orbitofrontal area (M = 2. 61 nA, SE = 0.55), compared to Right Orbitofrontal (M = 1.57 nA, SE = 0.29), Left Limbic (M = 1.31 nA, SE = 0.29), Right Limbic (M = 1.24 nA, SE = 0. 21), left fusiform gyrus (M = 2.23 nA, SE = 0.55), right fusiform gyrus (M = 2.29 nA, SE = 0.52), left temporal (M = 1.90 nA, SE = 0.38), and left occipital (M = 2.17 nA, SE = 0.39) areas. *Post-hoc* analysis also showed that the left limbic ROI (M = 1.57 nA, SE = 0.25) was more active during the emotional states “fear” than “sadness” and “happiness”. During the “joy” emotion condition, the Right Occipital area (M = 3.70 nA, SE = 0.57) demonstrated the highest activation, while the Right Limbic area (M = 0.94 nA, SE = 0.12) exhibited the lowest activation. Additionally, the Right Orbitofrontal area (M = 2.66 nA, SE = 0.56) showed the third-highest activation and was significantly different from the Left Limbic (M = 1.31 nA, SE = 0.27), Right Limbic (M = 0.94 nA, SE = 0.12), and Right Occipital (M = 3.70 nA, SE = 0.57) in the “joy” condition. Most importantly, the right OBF area was more active during “joy” (M = 2.66 nA, SE = 0.56) than other emotional states.

In summary, several category-specific activations were found (in a BCI perspective), brain signals were larger in the right TEMPORAL cortex during sadness (M = 3.78 nA, SE = 0.68) than joyful (M = 3.45 nA, SE = 0.58) or fearful emotional states (M = 2.97 nA, SE = 0.45). Brain signals were stronger in the left LIMBIC area during fearful (M = 1.57 nA, SE = 0.25), than sad (M = 1.31 nA, SE = 0.29) or joyful states (M = 1.31 nA, SE = 0.27). Finally, brain signals were stronger in the right OBF cortex during joyful (M = 2.66 nA, SE = 0.56) than fearful (M = 2.21 nA, SE = 0.40) or sad states (M = 1.57 nA, SE = 0.29 p<0.05).

### Nonparametric tests

**Figure 4:**
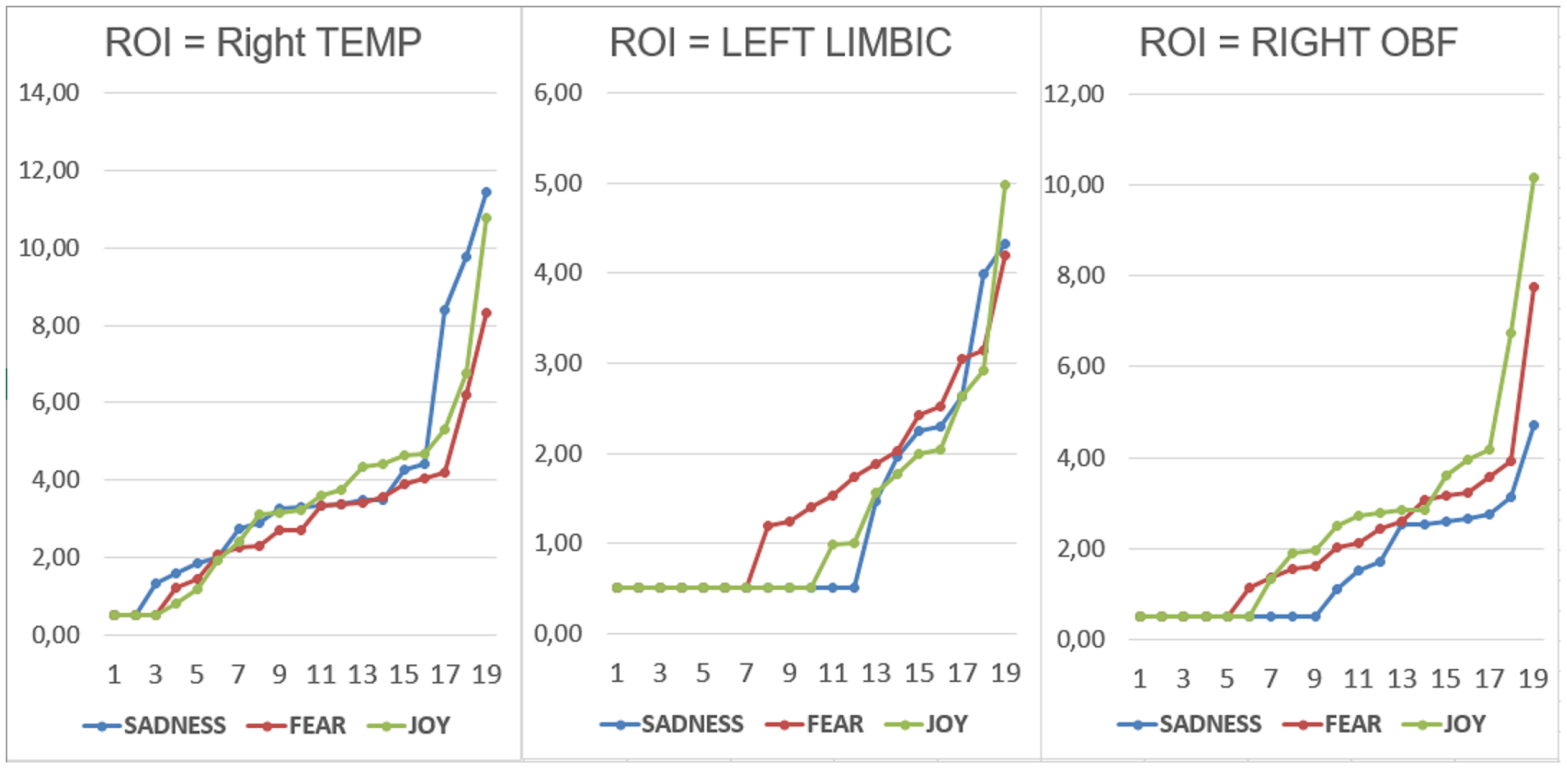
Individual data relative to dipole strengths recorded within the right temporal, left limbic and right orbitofrontal ROIs as a function of the emotional state felt.

Wilcoxon and Sign tests were used to further compare the magnitudes of brain signals recorded at the Left and Right Orbitofrontal, Left and Right Temporal, and Left and Right Limbic ROIs for each emotional state. The significance level was set at p < 0.05. The Wilcoxon test indicated that a significant difference was observed in the Right Temporal area between the “sadness” and “fear” conditions (Z = 3.21, p < 0.01) (**Figure 4**). Significant differences were also found in the Left Limbic area between the “fear” and “sadness” conditions (Z = 2.20, p < 0.05), and between the “fear” and “joy” conditions (Z = 2.20, p < 0.05). Furthermore, significant differences (p < 0.05) between the following pairs of variables in the Right Orbitofrontal area: “joy” and “sadness” (Z = 3.18, p < 0.01), and “joy” and “fear” (Z = 2.42, p < 0.05), as can be observed in **Figure 4**. The Sign test indicated significant differences in the Left Limbic area between the “fear” and “sadness” conditions (Z = 2.02, p < 0.05), and between the “fear” and “joy” conditions (Z = 2.60, p < 0.01). Additionally, a significant difference was observed in the Right Orbitofrontal area between the “joy” and “sadness” conditions (Z = 3.33, p < 0.001). In the Right Temporal area, a significant difference was found between the “sadness” and “fear” conditions (Z = 2.75, p = 0.01). The Right Temporal area exhibited higher activation during the “sadness” condition compared to other states. The Left Limbic area showed higher activation under the “fear” condition compared to the other two conditions. The Right Orbitofrontal area showed higher activation during the “joy” condition compared to other states.

### LORETA analysis

**Figure 5:**
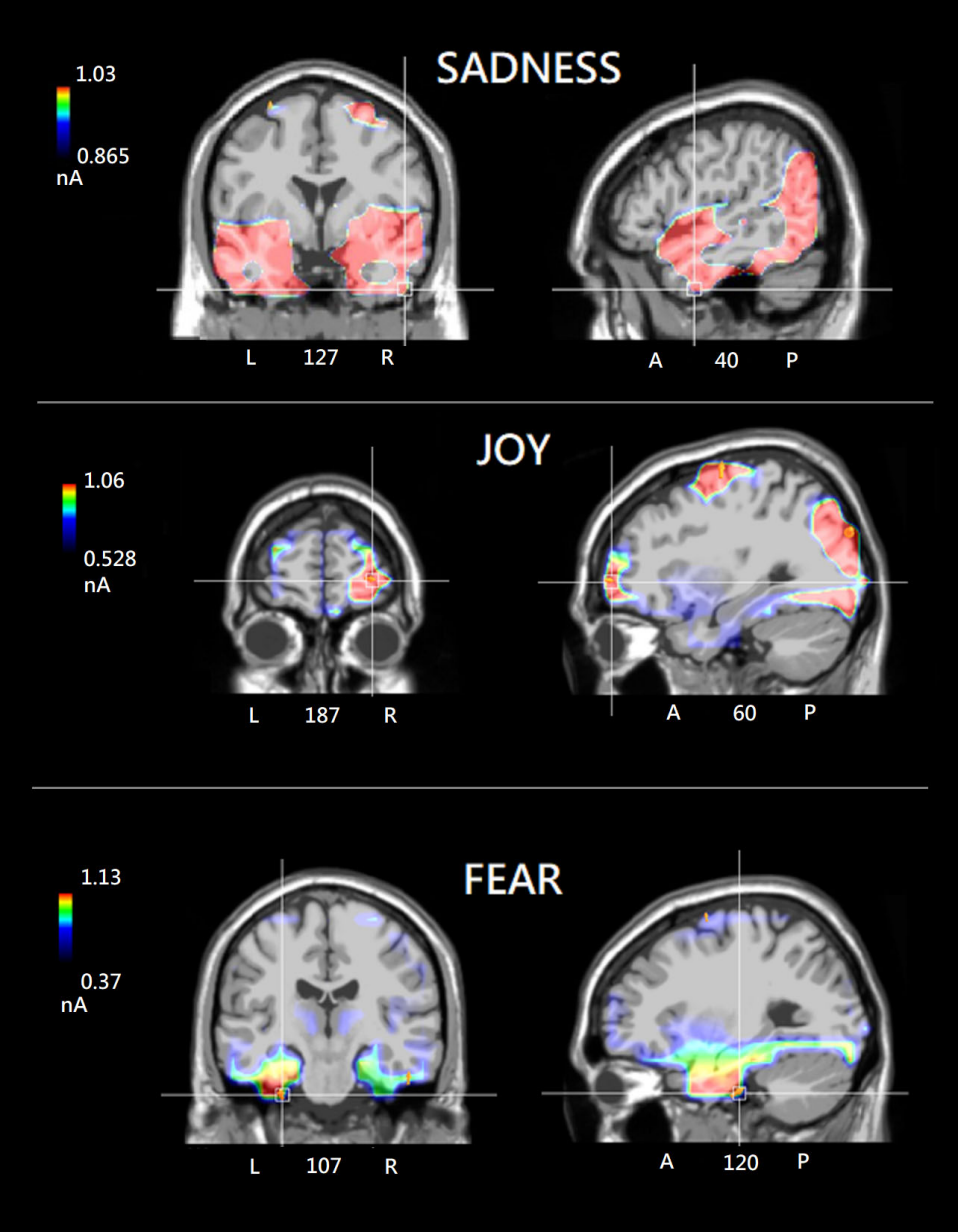
(Top) Group SwLORETA tomographic solutions from the nineteen participants included in the ANOVA analysis, focusing on the most active dipole in the right Temporal area under the “sadness” condition (N400: 400-600 ms). (Middle) Group SwLORETA tomographic solutions focusing on the most active dipole in the left Limbic area under the “fear” condition. (Bottom) Group SwLORETA tomographic solutions focusing on the most active dipole in the right Orbitofrontal region of interest under the “joy” condition. Obviously (due to inter-individual variability), the average inverse solutions do not exactly overlap with those of the individuals reported in the **Supplementary File**.

Individual and group swLORETA analyses were applied to electric signals recorded in the 400-600 ms time window (after probe onset), separately for each recalled emotional state. The individual source reconstruction solutions, i.e. list of active electromagnetic ROIs, can be found in **Supplementary File 1**. **Table 2** reports the list of strongest active sources found in the group analyses, while **Figure 5** depicts the neurometabolic changes in brain activation focused on coronal and sagittal views of the brain. The sagittal views are right-sided for sadness (as informed by numerical information about slice depth), and left-sided for fear. The strongest signals were recorded during the recalled emotional state of joy over the occipital ROI. This pattern fits with ERP amplitudes of N400 component that was larger during joy than fear and sadness emotional states. During recollection of sadness emotional state there was substantial activation of posterior visual areas, but especially of the right temporal and left frontal cortex. The fear emotional state was associated with a pronounced limbic activity, along with a reduced frontal involvement. During the joy emotional state, it was found a large occipital and FG activation along with a characteristic OBF involvement.

**Table 2:** Active electromagnetic dipoles (along with their Talairach coordinates) explaining brain voltage during the three recalled emotional states across the nineteen participants (Group analysis).

Magn.	T-x [mm]	T-y [mm]	T-z [mm]	HEM	LOBE	GYRUS	BA	ROI
“Sadness”								
1.892	40.9	-88.3	30	R	O	Middle Occipital	18	OCC
1.64	60.6	-55.0	-17.6	R	O	Fusiform	37	FUSIF
1.583	-58.5	-44.8	-16.9	L	T	Inferior Temporal	20	TEMP
1.549	-48.5	-76.2	-11.7	L	T	Fusiform	19	FUSIF
1.273	-28.5	-15.3	-29.6	L	Limbic	Uncus	20	LIMBIC
**1.172**	**31.0**	**91**	**-27.5**	**R**	**T**	**Superior Temporal**	**38**	**TEMP**
**1.107**	**50.8**	**-6**	**-28.2**	**R**	**T**	**Middle Temporal**	**21**	**TEMP**
1.033	50.8	-16.1	-22.2	R	T	Fusiform	20	FUSIF
**1.006**	**-28.5**	**-85**	**64.2**	**L**	**F**	**Superior Frontal**	**6**	**DPLF**
**0.8499**	**-85**	**64.4**	**16.8**	**L**	**F**	**Superior Frontal**	**10**	**OBF**
**0.8304**	**60.6**	**24**	**29.4**	**R**	**F**	**Precentral**	**6**	**DPLF**
“Fear”								
2.048	-48.5	-33.7	-23.6	L	T	Fusiform	20	FUSIF
**1.871**	**-18.5**	**-80**	**-28.9**	**L**	**Limbic**	**Uncus**	**36**	**LIMBIC**
**1.77**	**21.2**	**91**	**-27.5**	**R**	**Limbic**	**Uncus**	**38**	**LIMBIC**
**1.746**	**21.2**	**-24.5**	**-15.5**	**R**	**Limbic**	**Parahippocampal**	**35**	**LIMBIC**
1.677	-48.5	82	-20.0	L	T	Superior Temporal	38	TEMP
1.62	-58.5	-87	-21.5	L	T	Inferior Temporal	20	TEMP
1.443	31.0	-15.8	63.3	R	F	Precentral	6	DPLF
1.159	-85	-91.3	29.7	L	O	Cuneus	19	OCC
“Joy”								
2.652	31.0	-90.3	20.8	R	O	Middle Occipital	19	OCC
2.007	31.0	-15.8	63.3	R	F	Precentral	6	DPLF
**1.503**	**31.0**	**55.3**	**70**	**R**	**F**	**Middle Frontal**	**10**	**OBF**
**1.479**	**11.3**	**65.3**	**79**	**R**	**F**	**Superior Frontal**	**10**	**OBF**
**1.202**	**-38.5**	**43.4**	**23.9**	**L**	**F**	**Middle Frontal**	**10**	**OBF**
1.135	-85	-63.8	59.0	L	P	Superior Parietal	7	AIP

Magn., magnitude in nA; Hem, Hemisphere; BA, Brodmann areas. In bold are the key structures selected as most distinctive for a BCI application.

## Discussion

The study aimed to observe distinctive patterns of brain activation as participants recalled specific emotional states (evoked by pictograms). The study analyzed data from 20 healthy male and female participants to identify frequently occurring neural markers that could be detected in most or all of the participants. The goal was to decipher emotions from brainwaves, focusing on a Brain-Computer Interface perspective. Contrasting sources of reconstruction on an individual data level is notably novel, but this approach has been previously implemented in BCI research (e.g. 92–95). Furthermore, it is now possible to perform source reconstruction of EEG data during online acquisition, which makes a BCI approach even more practical (96, 97).

From a neuroscientific perspective, one of the most significant findings of this study was the pronounced activation of the right hemisphere compared to the left hemisphere during recollection of all emotional states. This was particularly evident over the posterior brain areas, suggesting a key role for these regions in the vividness and visual components of emotional experience recall. Previous studies have consistently reported a right hemispheric asymmetry for visuomotor imagery (98), spatial navigation (99), emotions (100), and music imagery (101–103). The strong activation that was observed over the right temporal lobe across all three emotional conditions, as highlighted by Liu et al. (104), might be related to the specific affective nature of the emotional states, and to the presence of imagined social content such as people or faces.

### Joy

Overall, visual brain areas were most active during “joy” emotional condition (as found by 105), thus resulting in stronger electromagnetic signals. This piece of evidence fits with the findings of larger N400 mean area amplitude values recorded to joy than other emotional states in the related ERP study (20). This suggests how joyful states might be associated with more vivid, lively, or energetic brain signals. Coherent with this interpretation were the findings of enhanced anterior prefrontal (OBF) cortex specifically during positive emotional states, which is linked to its role in the dopaminergic reward circuitry, as reported by various studies (43–46). This text demonstrates the similarities between the concepts of happiness as pleasure, cheerfulness, and positive mood. The experience of happiness and cheerfulness is thought to be closely linked to the Orbitofrontal cortex (72). Furthermore, our data showed an asymmetry in the OBF activation, with a slightly more pronounced activation over the right orbitofrontal cortex, possibly related to the imagery nature of recalled affective states. Intriguingly a neurological study found that lesions over the right OBF cortex were related to impaired emotional recognition of facial expressions for happiness (106).

### Fear

The present findings showed an enhanced activity of the limbic area during “fear” affective states. The limbic system, that was found here more active over the left hemisphere, includes regions like the amygdala and thalamus, which are linked to processing stimulus emotional significance and arousal (32, 55–58). Limbic structures are also thought to be involved in encoding the emotional value of experiences (35). Indeed, numerous studies on fear (53, 107) have consistently emphasized the pivotal role of the amygdala and the broader Limbic system in experiencing a range of emotions beyond fear. The Limbic system is intricately interconnected with the “emotional brain,” as proposed by Pessoa (108), and has been consistently observed to be active in the psychological experience of fear, both in humans and animals (49). This supports the significance of this neuro-marker as a reliable signature of felt fearful state. The second more distinctive feature of fearful state, in this study, was the frontal de-activation, with smaller brain signals coming from the frontal cortex in most of the participants. This evidence fits with the major role of the prefrontal cortex in fear- control, extinction and regulation (109, 110).

### Sadness

In the “sadness” condition, it was observed the most significant activation of the right temporal ROI, which aligns perfectly with the existing literature. The study found notable activity in the right superior temporal gyrus, which is known for its role in perceiving facial expressions of emotion, indicating that the right hemisphere may exhibit increased activity during experiences of sadness (70, 71). A study by Proverbio and colleagues (69) coherently reported significant activation of the right middle temporal gyrus when participants were exposed to stimuli inducing negative emotions (such as sad music), further highlighting the role of this region in processing negative emotional cues. The right temporal cortex is also found more active in depressed patients (63) and is thought to be at the root of the ability to perceive negative emotions, depression and sadness. Relatedly, a recent meta-analysis on brain network features specific to sadness reported how the right temporal area was associated with negative emotions and sadness (83). According to Adolphs et al. (111) the right temporoparietal cortex is important in processing negative emotional facial expressions. Furthermore, the temporal gyrus is known to be active during negative emotional mood, such as depression and anxiety disorders (71), Sugiura et al. (70).

Overall, this research has provided valuable data for the analysis and study of neuro-markers derived from EEG localization. Based on these principles, classifiers could be developed to identify the emotional state of a patient with LIS, even when unconscious. As for whether LORETA can accurately estimate sources far from the scalp surface, such as the amygdala, thalamus, and limbic system, there is much evidence in the literature. A recent study using high-density (256-channel) scalp EEG (recorded simultaneously with intracranial local field potentials from deep brain structures in patients undergoing deep brain stimulation) demonstrated that EEG source localization was able to detect and correctly localize spontaneous alpha activity generated in the thalamus (112). Again, Seeber and coauthors (113) placed deep brain stimulation (DBS) electrodes in centromedial thalamus and accumbens nuclei providing the unique opportunity to record subcortical activity simultaneously with high-density scalp EEG. Indeed, in his review, Lopes da Silva (114) conclusively concluded that subcortical Local Field Potentials can reach the scalp EEG by volume conduction, and that high-resolution EEG scalp recordings can be used to estimate corresponding sources localized in deep subcortical brain areas. In fact, Cebolla et al. (115) using swLORETA source reconstruction found thalamic and cerebellar generators for motor imagery, by localizing scalp recorded EEG, while Gerez et al. (116) and Suzuki and Kirino (117) found LORETA evidences of amygdala activity in combined EEG and fMRI studies on schizophrenics and patients affected by panic disorder.

In summary, scalp-recorded EEG/ERP signals were here source reconstructed to generate recognizable patterns of recalled sadness, joy and fear emotional states in a group of 20 participants in a BCI perspective. Regularities were found in the localization and relative strength of intracranial neural sources depending on the emotional state of interest, despite obvious individual differences. The main features observed were widespread right temporal activity associated during recollection of sadness, combined with left frontal hyperactivity (rumination); more pronounced left limbic activation, combined with clear frontal disengagement (uncontrolled emotional response) during recollection of fear; and more vivid and lively visual activity, combined with activation of the reward OBF circuit for the positive emotional state of cheerfulness and joy.

By analyzing brain activation signals, it may be possible in the future to detect and classify the internal states and desires of patients, even in cases where they are unable to communicate or express their needs. This could significantly improve their quality of life and help address communication challenges often faced by individuals with coma or locked-in syndrome. Research has also shown that imagination can elicit comparable responses to emotional stimuli (22), providing additional support for the potential of mind-reading approaches in BCI systems. Overall, the findings suggest that using mind reading techniques in BCI systems (14, 118–120) could significantly benefit individuals with consciousness disorders, enabling social communication. Incommunicability can lead to the loss of one’s social role, social isolation, and the inability to benefit from others’ compassion, affection, and empathy.

## Study limits

One potential limitation of this study is the relatively small sample size; therefore, future research should aim to investigate larger samples. However, most of the sources identified were active in 100% of participants (see individual dipole lists in **Supplementary File 1**), albeit with some hemispheric differences, supporting the robustness of the data and the generalizability of the results. A further potential limitation might come from the fact that the recalled affective states were to be voluntarily activated, and did not derive from current circumstantial real events. This condition may not fully correspond to people’s experiences in real situations related to such needs, but the same criticality holds for any study involving imagery paradigms. One key concern is that probes might evoke a blend of emotions rather than discrete ones. Emotions in real-life situations are often complex, making it challenging to attribute observed brain activity solely to a specific emotion. Additionally, the study’s reliance on the recall of imagination of emotional stimuli may not fully capture the multifaceted nature of emotional experiences (55). Real emotions involve a complex interplay of thoughts, bodily sensations, and subjective feelings. Research in neuroscience that suggests imagination is a bit like a less vivid and detailed version of our regular sensory experiences, since the data is quite noisy (79).

## Data availability statement

The original contributions presented in the study are included in the article/**Supplementary Material**. Further inquiries can be directed to the corresponding author.

## Ethics statement

The studies involving humans were approved by Ethics Committee of University of Milano-Bicocca (protocol no: RM-2020-242). The studies were conducted in accordance with the local legislation and institutional requirements. The participants provided their written informed consent to participate in this study. Written informed consent was obtained from the individual(s) for the publication of any potentially identifiable images or data included in this article.

## Author contributions

AMP: Data curation, Formal analysis, Funding acquisition, Investigation, Resources, Supervision, Validation, Visualization, Writing – original draft, Writing – review & editing. FC: Data curation, Formal analysis, Investigation, Methodology, Visualization, Writing – original draft.
