# Supplementary material for: Neural correlates of recalled sadness, joy, and fear states: a source reconstruction EEG study
Source: Front Psychiatry. 2024 Apr 4;15:1357770. doi: 10.3389/fpsyt.2024.1357770 (PMC11024723; doi:10.3389/fpsyt.2024.1357770)
Supplement: Supplementary file 1 [file DataSheet_1.pdf]

Supplementary file

Below are the individual swLORETA reconstructions for the emotional state "sadness".

06SC	SADNESS								
Dipole No.	Magnitude E-06	T-x [mm]	T-y [mm]	T-z [mm]	Hem	Lobe	Gyrus	BA	ROI
172	4.241	60.6	-55	-17.6	R	O	Fusiform Gyrus	37	FUSIF
439	4.023	-58	-23.6	-15	L	T	Middle temporal Gyrus	21	TEMP
760	3.591	2.1	-1.5	64.7	R	F	Superior Frontal Gyrus	6	DPLF
625	3.492	50.8	-1.5	-27.5	R	T	Middle Temporal Gyrus	21	TEMP
19	3.435	-28.5	-97.6	-5.7	L	O	Lingual Gyrus	18	OCC
1049	3.160	-28.5	54	16	L	F	Superior Frontal Gyrus	10	OBF
471	2.993	31	-15	-29.6	R	Limbic	Uncus	20	LIMBIC
141	2.982	2.1	-73	48.5	R	P	Precuneus	7	AIP

07AM	SADNESS								
Dipole No.	Magnitude E-06	T-x [mm]	T-y [mm]	T-z [mm]	Hem	Lobe	Gyrus	BA	ROI
448	9.785	71.2	-17.6	-7.1	R	T	Middle temporal Gyrus	21	TEMP
929	8.117	-48.5	22	31	L	F	Middle Frontal Gyrus	9	DPLF
242	7.785	-59	-55	-17.6	L	T	Fusiform Gyrus	37	FUSIF
873	7.318	-48.5	16	-4.8	L	F	Inferior Frontal Gyrus	47	OBF
768	5.776	-18.5	8.8	-27.5	L	T	Superior Temporal Gyrus	38	TEMP
732	3.696	40.9	-0.4	56	R	F	Middle Frontal Gyrus	6	DPLF
99	2.679	-28.5	-81.9	40	L	P	Precuneus	19	AIP

08MS	SADNESS								
Dipole No.	Magnitude E-06	T-x [mm]	T-y [mm]	T-z [mm]	Hem	Lobe	Gyrus	BA	ROI
111	7.284	50.8	-68	4.9	R	O	Middle Occipital Gyrus	19	OCC
164	2.766	-38.5	-72	40	L	P	Precuneus	19	AIP
863	2.544	-28.5	10	56.8	L	F	Middle Frontal Gyrus	6	DPLF
557	1.964	21.2	-8	-28.5	R	Limbic	Uncus	36	LIMBIC
896	1.962	21.2	20	58	R	F	Superior Frontal Gyrus	6	DPLF
1047	1.548	-28.5	56	-2.1	L	F	Superior Frontal Gyrus	10	OBF

09AF	SADNESS								
Dipole No.	Magnitude E-06	T-x [mm]	T-y [mm]	T-z [mm]	Hem	Lobe	Gyrus	BA	ROI
106	2.720	-48.5	-76.2	-11.7	L	T	Fusiform Gyrus	19	FUSIF
1026	2.367	31	55.3	7.1	R	F	Middle Frontal Gyrus	10	OBF
11	2.073	2.1	-99	11.4	R	O	Cuneus	18	OCC
625	1.897	50.8	-1.5	-27.5	R	T	Middle Temporal Gyrus	21	TEMP
172	1.706	60.6	-55	-17.6	R	O	Fusiform Gyrus	37	FUSIF
802	1.634	-59	4.9	2.7	L	T	Superior Temporal Gyrus	22	TEMP
979	1.629	-48.5	34.9	4.9	L	F	Inferior Frontal Gyrus	45	OBF
47	1.583	-8.5	-91.3	29.7	L	O	Cuneus	19	OCC
774	1.553	-18.5	-1.5	64.7	L	F	Superior Frontal Gyrus	6	DPLF
487	1.549	2.1	-23	62	R	F	Medial Frontal Gyrus	6	DPLF
241	1.547	-48.5	-62	40.9	L	P	Inferior Parietal Lobule	39	AIP

10LD	SADNESS								
Dipole No.	Magnitude E-06	T-x [mm]	T-y [mm]	T-z [mm]	Hem	Lobe	Gyrus	BA	ROI
625	3.378	50.8	-1.5	-27.5	R	T	Middle Temporal Gyrus	21	TEMP
242	3.228	-59	-55	-17.6	L	T	Fusiform Gyrus	37	FUSIF
878	2.834	-48.5	11.4	39	L	F	Middle Frontal Gyrus	8	DPLF
807	2.608	60.6	13.3	21.2	R	F	Inferior Frontal Gyrus	45	OBF
665	2.254	-8.5	-1.5	-27.5	L	Limbic	Uncus	28	LIMBIC
51	1.462	-18.5	-91.3	29.7	L	O	Cuneus	19	OCC
7	1.316	11.4	-97.6	2.1	R	O	Cuneus	18	OCC
1056	1.289	-8.5	64.4	16.8	L	F	Superior Frontal Gyrus	10	OBF
73	1.155	21.2	-81.9	40	R	P	Precuneus	19	AIP
409	0.958	2.1	-28.5	26	R	Limbic	Cingulate Gyrus	23	LIMBIC

11SG	SADNESS								
Dipole No.	Magnitude E-06	T-x [mm]	T-y [mm]	T-z [mm]	Hem	Lobe	Gyrus	BA	ROI
318	2.975	60.6	-41.5	42.9	R	P	Inferior Parietal Lobule	40	AIP
986	2.753	40.9	42.9	24.2	R	F	Middle Frontal Gyrus	10	OBF
993	2.483	21.2	40.9	42.9	R	F	Superior Frontal Gyrus	8	DPLF
242	2.225	-59	-55	-17.6	L	T	Fusiform Gyrus	37	FUSIF
20	2.201	40.9	-85.8	-11.7	R	O	Inferior Occipital Gyrus	18	OCC
12	1.966	-8	-96.5	-13	L	O	Lingual Gyrus	17	OCC
724	1.576	40.9	8.8	-27.5	R	T	Superior Temporal Gyrus	38	TEMP
979	1.326	-48.5	34.9	4.9	L	F	Inferior Frontal Gyrus	45	OBF
945	1.242	11.4	34.9	4.9	R	Limbic	Anterior Cingulate	24	LIMBIC
99	1.126	-28.5	-81.9	40	L	P	Precuneus	19	AIP

12AC	SADNESS								
Dipole No.	Magnitude E-06	T-x [mm]	T-y [mm]	T-z [mm]	Hem	Lobe	Gyrus	BA	ROI
619	8.399	60.6	-2.9	-5.7	R	T	Middle Temporal Gyrus	21	TEMP
595	7.502	-28.5	-15.8	63	L	F	Precentral Gyrus	6	DPLF
65	6.283	40.9	-81	31	R	O	Superior Occipital Gyrus	19	OCC
109	5.524	50.8	-66.1	-11.3	R	T	Fusiform Gyrus	19	FUSIF
665	3.986	-8.5	-1.5	-27.5	L	Limbic	Uncus	28	LIMBIC
563	3.663	21.2	-15.8	63	R	F	Precentral Gyrus	6	DPLF
702	3.342	-59	-1.5	-21	L	T	Middle temporal Gyrus	21	TEMP
1056	2.734	-8.5	64.4	16.8	L	F	Superior Frontal Gyrus	10	OBF
219	2.442	-8.5	-63.8	58.8	L	P	Superior Parietal Lobule	7	AIP

14LD	SADNESS								
Dipole No.	Magnitude E-06	T-x [mm]	T-y [mm]	T-z [mm]	Hem	Lobe	Gyrus	BA	ROI
801	5.130	-48.5	-0.4	47	L	F	Precentral Gyrus	6	DPLF
1	4.618	31	-97.6	-5.7	R	O	Inferior Occipital Gyrus	18	OCC
242	4.586	-59	-55	-17.6	L	T	Fusiform Gyrus	37	FUSIF
959	3.216	2.1	29.7	58.8	R	F	Superior Frontal Gyrus	6	DPLF
164	3.164	-38.5	-72	40	L	P	Precuneus	19	AIP
694	2.750	-48.5	-1.5	-27.5	L	T	Inferior Temporal Gyrus	20	TEMP
526	2.734	60.6	-8.5	-21.7	R	T	Inferior Temporal Gyrus	20	TEMP
1056	1.399	-8.5	64.4	16.8	L	F	Superior Frontal Gyrus	10	OBF

15CC	SADNESS								
Dipole No.	Magnitude E-06	T-x [mm]	T-y [mm]	T-z [mm]	Hem	Lobe	Gyrus	BA	ROI
978	3.526	-48.5	36	-2.9	L	F	Middle Frontal Gyrus	47	OBF
172	3.517	60.6	-55	-17.6	R	O	Fusiform Gyrus	37	FUSIF
625	3.323	50.8	-1.5	-27.5	R	T	Middle Temporal Gyrus	21	TEMP
1007	3.131	2.1	40.9	50.8	R	F	Superior Frontal Gyrus	8	DPLF
108	2.823	-48.5	-77.6	4.3	L	O	Middle Occipital Gyrus	19	OCC
70	2.488	31	-81.9	40	R	P	Precuneus	19	AIP
242	2.139	-59	-55	-17.6	L	T	Fusiform Gyrus	37	FUSIF
11	2.069	2.1	-99	11.4	R	O	Cuneus	18	OCC
1024	1.513	40.9	55.3	7.1	R	F	Middle Frontal Gyrus	10	OBF
665	1.467	-8.5	-1.5	-27.5	L	Limbic	Uncus	28	LIMBIC
483	1.135	2.1	-20	27	R	Limbic	Cingulate Gyrus	23	LIMBIC

16MN	SADNESS								
Dipole No.	Magnitude E-06	T-x [mm]	T-y [mm]	T-z [mm]	Hem	Lobe	Gyrus	BA	ROI
175	11.450	60.6	-58	5.9	R	T	Middle Temporal Gyrus	21	TEMP
1014	10.060	-18.5	40.9	42.9	L	F	Superior Frontal Gyrus	8	DPLF
1056	9.849	-8.5	64.4	16.8	L	F	Superior Frontal Gyrus	10	OBF
253	9.484	60.6	-51	33	R	P	Supramarginal Gyrus	40	AIP
172	8.507	60.6	-55	-17.6	R	O	Fusiform Gyrus	37	FUSIF
990	8.051	31	42.9	33	R	F	Middle Frontal Gyrus	9	DPLF
20	7.112	40.9	-85.8	-11.7	R	O	Inferior Occipital Gyrus	18	OCC
242	6.224	-59	-55	-17.6	L	T	Fusiform Gyrus	37	FUSIF
937	4.723	31	36.6	-11.3	R	F	Middle Frontal Gyrus	11	OBF
665	4.326	-8.5	-1.5	-27.5	L	Limbic	Uncus	28	LIMBIC
51	4.075	-18.5	-91.3	29.7	L	O	Cuneus	19	OCC
438	1.595	-48.5	-32	53	L	P	Inferior Parietal Lobule	40	AIP

18MC	SADNESS								
**Dipole No.**	**Magnitude E-06**	**T-x [mm]**	**T-y [mm]**	**T-z [mm]**	**Hem**	**Lobe**	**Gyrus**	**BA**	**ROI**
449	4.396	60.6	-15.8	-21.7	R	T	Inferior Temporal Gyrus	20	TEMP
318	3.645	60.6	-41.5	42.9	R	P	Inferior Parietal Lobule	40	AIP
624	2.850	60.6	-5.7	36.6	R	F	Precentral Gyrus	6	DPLF
665	2.632	-8.5	-1.5	-27.5	L	Limbic	Uncus	28	LIMBIC
439	2.520	-58	-23.6	-15	L	T	Middle temporal Gyrus	21	TEMP
36	2.173	11.4	-91.3	29.7	R	O	Cuneus	19	OCC
108	2.029	-48.5	-77.6	4.3	L	O	Middle Occipital Gyrus	19	OCC
1047	1.882	-28.5	56	-2.1	L	F	Superior Frontal Gyrus	10	OBF
272	1.622	11.4	-49.5	24.2	R	Limbic	Cingulate Gyrus	31	LIMBIC

19MR	SADNESS								
**Dipole No.**	**Magnitude E-06**	**T-x [mm]**	**T-y [mm]**	**T-z [mm]**	**Hem**	**Lobe**	**Gyrus**	**BA**	**ROI**
1048	3.597	-28.5	55.3	7.1	L	F	Middle Frontal Gyrus	10	OBF
1025	2.666	31	56	-2.1	R	F	Superior Frontal Gyrus	10	OBF
319	2.121	50.8	-33.7	-23.6	R	T	Fusiform Gyrus	20	FUSIF
556	2.066	31	-15.8	63	R	F	Precentral Gyrus	6	DPLF
22	2.045	40.9	-88.3	2.7	R	O	Middle Occipital Gyrus	18	OCC
471	2.031	31	-15	-29.6	R	Limbic	Uncus	20	LIMBIC
445	2.008	-59	-29.6	34.9	L	P	Inferior Parietal Lobule	40	AIP
449	2.002	60.6	-15.8	-21.7	R	T	Inferior Temporal Gyrus	20	TEMP
134	1.331	11.4	-73	48.5	R	P	Precuneus	7	AIP

21GC	SADNESS								
**Dipole No.**	**Magnitude E-06**	**T-x [mm]**	**T-y [mm]**	**T-z [mm]**	**Hem**	**Lobe**	**Gyrus**	**BA**	**ROI**
556	4.228	31	-15.8	63	R	F	Precentral Gyrus	6	DPLF
22	3.694	40.9	-88.3	2.7	R	O	Middle Occipital Gyrus	18	OCC
18	1.438	-18.5	-99	2.1	L	O	Cuneus	18	OCC
242	1.350	-59	-55	-17.6	L	T	Fusiform Gyrus	37	FUSIF
526	1.335	60.6	-8.5	-21.7	R	T	Inferior Temporal Gyrus	20	TEMP
921	1.304	-38.5	27	-11.3	L	F	Inferior Frontal Gyrus	47	OBF
241	1.145	-48.5	-62	40.9	L	P	Inferior Parietal Lobule	39	AIP
694	1.131	-48.5	-1.5	-27.5	L	T	Inferior Temporal Gyrus	20	TEMP
999	1.108	2.1	48	-16.9	R	F	Medial Frontal Gyrus	11	OBF

23FN	SADNESS								
Dipole No.	Magnitude E-06	T-x [mm]	T-y [mm]	T-z [mm]	Hem	Lobe	Gyrus	BA	ROI
57	4.594	-38.5	-85.8	-11.7	L	O	Inferior Occipital Gyrus	18	OCC
190	3.633	40.9	-63	49.5	R	P	Superior Parietal Lobule	7	AIP
172	3.567	60.6	-55	-17.6	R	O	Fusiform Gyrus	37	FUSIF
982	3.373	50.8	45	5.9	R	F	Middle Frontal Gyrus	46	DPLF
806	3.137	60.6	14	13.3	R	F	Inferior Frontal Gyrus	44	OBF
27	3.002	31	-90.3	21.2	R	O	Middle Occipital Gyrus	19	OCC
233	1.998	-38.5	-63	49.5	L	P	Superior Parietal Lobule	7	AIP
582	1.954	-18.5	-8	-28.5	L	Limbic	Uncus	36	LIMBIC
792	1.439	-38.5	-0.4	56	L	F	Middle Frontal Gyrus	6	DPLF
945	1.247	11.4	34.9	4.9	R	Limbic	Anterior Cingulate	24	LIMBIC

25DE	SADNESS								
Dipole No.	Magnitude E-06	T-x [mm]	T-y [mm]	T-z [mm]	Hem	Lobe	Gyrus	BA	ROI
704	3.688	-59	-2.9	-5.7	L	T	Middle temporal Gyrus	21	TEMP
166	3.643	-48.5	-66.1	-11.3	L	T	Fusiform Gyrus	19	FUSIF
625	3.308	50.8	-1.5	-27.5	R	T	Middle Temporal Gyrus	21	TEMP
319	3.205	50.8	-33.7	-23.6	R	T	Fusiform Gyrus	20	FUSIF
471	2.759	31	-15	-29.6	R	Limbic	Uncus	20	LIMBIC
848	2.626	-8.5	18	-18.5	L	F	Rectal Gyrus	11	OBF
141	2.112	2.1	-73	48.5	R	P	Precuneus	7	AIP
164	2.106	-38.5	-72	40	L	P	Precuneus	19	AIP
1014	1.833	-18.5	40.9	42.9	L	F	Superior Frontal Gyrus	8	DPLF
1007	1.601	2.1	40.9	50.8	R	F	Superior Frontal Gyrus	8	DPLF

26MP	SADNESS								
Dipole No.	Magnitude E-06	T-x [mm]	T-y [mm]	T-z [mm]	Hem	Lobe	Gyrus	BA	ROI
64	3.254	40.9	-80	22	R	T	Middle Temporal Gyrus	19	TEMP
685	2.663	-28.5	-8.5	64.4	L	F	Superior Frontal Gyrus	6	DPLF
57	2.401	-38.5	-85.8	-11.7	L	O	Inferior Occipital Gyrus	18	OCC
933	2.161	50.8	33	23.1	R	F	Middle Frontal Gyrus	46	DPLF
219	1.921	-8.5	-63.8	58.8	L	P	Superior Parietal Lobule	7	AIP
389	1.189	50.8	-32	53	R	P	Inferior Parietal Lobule	40	AIP

28FR	SADNESS								
Dipole No.	Magnitude E-06	T-x [mm]	T-y [mm]	T-z [mm]	Hem	Lobe	Gyrus	BA	ROI
20	3.793	40.9	-85.8	-11.7	R	O	Inferior Occipital Gyrus	18	OCC
595	3.340	-28.5	-15.8	63	L	F	Precentral Gyrus	6	DPLF
454	2.958	60.6	-20	18	R	P	Postcentral Gyrus	40	AIP
113	2.872	50.8	-70	23.1	R	T	Middle Temporal Gyrus	39	TEMP
879	2.705	-59	14	13.3	L	F	Inferior Frontal Gyrus	44	OBF
807	2.523	60.6	13.3	21.2	R	F	Inferior Frontal Gyrus	45	OBF
793	2.360	-48.5	8	-20	L	T	Superior Temporal Gyrus	38	TEMP
582	2.290	-18.5	-8	-28.5	L	Limbic	Uncus	36	LIMBIC
1007	1.817	2.1	40.9	50.8	R	F	Superior Frontal Gyrus	8	DPLF
219	1.752	-8.5	-63.8	58.8	L	P	Superior Parietal Lobule	7	AIP

29EM	SADNESS								
Dipole No.	Magnitude E-06	T-x [mm]	T-y [mm]	T-z [mm]	Hem	Lobe	Gyrus	BA	ROI
242	6.198	-59	-55	-17.6	L	T	Fusiform Gyrus	37	FUSIF
15	5.793	-8	-99	11.4	L	O	Cuneus	18	OCC
1	4.696	31	-97.6	-5.7	R	O	Inferior Occipital Gyrus	18	OCC
1046	4.329	-18.5	52	34	L	F	Superior Frontal Gyrus	9	DPLF
624	2.659	60.6	-5.7	36.6	R	F	Precentral Gyrus	6	DPLF
450	1.847	60.6	-16.9	-15	R	T	Inferior Temporal Gyrus	20	TEMP
1026	1.695	31	55.3	7.1	R	F	Middle Frontal Gyrus	10	OBF

30CG	SADNESS								
Dipole No.	Magnitude E-06	T-x [mm]	T-y [mm]	T-z [mm]	Hem	Lobe	Gyrus	BA	ROI
50	4.279	-18.5	-90.3	21.2	L	O	Cuneus	18	OCC
64	4.278	40.9	-80	22	R	T	Middle Temporal Gyrus	19	TEMP
647	3.802	31	-8.5	64.4	R	F	Precentral Gyrus	6	DPLF
172	3.499	60.6	-55	-17.6	R	O	Fusiform Gyrus	37	FUSIF
1051	2.516	11.4	64.7	8	R	F	Superior Frontal Gyrus	10	OBF
219	2.480	-8.5	-63.8	58.8	L	P	Superior Parietal Lobule	7	AIP
611	1.903	-59	-8.5	-14	L	T	Inferior Temporal Gyrus	21	TEMP
970	1.456	-18.5	31	49.5	L	F	Superior Frontal Gyrus	8	DPLF

31AC	SADNESS								
Dipole No.	Magnitude E-06	T-x [mm]	T-y [mm]	T-z [mm]	Hem	Lobe	Gyrus	BA	ROI
172	4.243	60.6	-55	-17.6	R	O	Fusiform Gyrus	37	FUSIF
439	4.021	-58	-23.6	-15	L	T	Middle temporal Gyrus	21	TEMP
760	3.590	2.1	-1.5	64.7	R	F	Superior Frontal Gyrus	6	DPLF
625	3.490	50.8	-1.5	-27.5	R	T	Middle Temporal Gyrus	21	TEMP
19	3.428	-28.5	-97.6	-5.7	L	O	Lingual Gyrus	18	OCC
1049	3.162	-28.5	54	16	L	F	Superior Frontal Gyrus	10	OBF
471	2.985	31	-15	-29.6	R	Limbic	Uncus	20	LIMBIC
141	2.976	2.1	-73	48.5	R	P	Precuneus	7	AIP
5	2.761	11.4	-97	-13	R	O	Lingual Gyrus	17	OCC

Below are the individual swLORETA reconstructions for the emotional state "fear".

06SC FEAR

Dipole No.	Magnitude E-06	T-x [mm]	T-y [mm]	T-z [mm]	Hem	Lobe	Gyrus	BA	ROI
30	5.565	21.2	-91.3	29.7	R	O	Cuneus	19	OCC
109	4.739	50.8	-66.1	-11.3	R	T	Fusiform Gyrus	19	FUSIF
625	3.423	50.8	-1.5	-27.5	R	T	Middle Temporal Gyrus	21	TEMP
740	3.338	21.2	8.8	-27.5	R	Limbic	Uncus	38	LIMBIC
950	3.150	2.1	38	-17.6	R	F	Medial Frontal Gyrus	11	OBF
665	3.148	-8.5	-1.5	-27.5	L	Limbic	Uncus	28	LIMBIC
50.8	2.889	-18.5	-91.3	29.7	L	O	Cuneus	19	OCC
970	2.394	-18.5	31	49.5	L	F	Superior Frontal Gyrus	8	DPLF
940	2.114	31	31	40.9	R	F	Middle Frontal Gyrus	8	DPLF
446	1.516	-59	-31	43.6	L	P	Inferior Parietal Lobule	40	AIP

07AM FEAR

Dipole No.	Magnitude E-06	T-x [mm]	T-y [mm]	T-z [mm]	Hem	Lobe	Gyrus	BA	ROI
610	7.232	-59	-8.5	-21.7	L	T	Inferior Temporal Gyrus	20	TEMP
982	5.564	50.8	44.6	5.9	R	F	Middle Frontal Gyrus	46	DPLF
242	5.305	-59	-55	-17.6	L	T	Fusiform Gyrus	37	FUSIF
172	4.860	60.6	-55	-17.6	R	O	Fusiform Gyrus	37	FUSIF
177	3.364	60.6	-59.5	23.1	R	T	Superior Temporal Gyrus	39	TEMP
27	3.326	31	-90.3	21.2	R	O	Middle Occipital Gyrus	19	OCC
685	3.212	-28.5	-8.5	64.4	L	F	Superior Frontal Gyrus	6	DPLF
149	2.821	-8.5	-73	48.5	L	P	Precuneus	7	AIP
126	2.691	21.2	-73	48.5	R	P	Precuneus	7	AIP

08MS FEAR

Dipole No.	Magnitude E-06	T-x [mm]	T-y [mm]	T-z [mm]	Hem	Lobe	Gyrus	BA	ROI
22	8.922	40.9	-88.3	2.7	R	O	Middle Occipital Gyrus	18	OCC
624	7.607	60.6	-5.7	36.6	R	F	Precentral Gyrus	6	DPLF
167	5.503	-48.5	-67	-4	L	O	Middle Occipital Gyrus	37	OCCIPITAL
198	4.484	21.2	-63.8	58.8	R	P	Superior Parietal Lobule	7	AIP
625	4.045	50.8	-1.5	-27.5	R	T	Middle Temporal Gyrus	21	TEMP
149	3.519	-8.5	-73	48.5	L	P	Precuneus	7	AIP
1047	3.227	-28.5	56	-2.1	L	F	Superior Frontal Gyrus	10	OBF
863	3.101	-28.5	10	56.8	L	F	Middle Frontal Gyrus	6	DPLF
497	3.044	-28.5	-15	-29.6	L	Limbic	Uncus	20	LIMBIC

09AF	FEAR								
Dipole No.	Magnitude E-06	T-x [mm]	T-y [mm]	T-z [mm]	Hem	Lobe	Gyrus	BA	ROI
982	5.260	50.8	44.6	5.9	R	F	Middle Frontal Gyrus	46	DPLF
802	3.371	-59	4.9	2.7	L	T	Superior Temporal Gyrus	22	TEMP
106	3.284	-48.5	-76.2	-11.7	L	T	Fusiform Gyrus	19	FUSIF
172	2.653	60.6	-55	-17.6	R	O	Fusiform Gyrus	37	FUSIF
21.2	2.266	40.9	-87	-4.8	R	O	Inferior Occipital Gyrus	18	OCC
792	2.097	-38.5	-0.4	56	L	F	Middle Frontal Gyrus	6	DPLF
318	1.684	60.6	-41.5	42.9	R	P	Inferior Parietal Lobule	40	AIP
164	1.425	-38.5	-72	39.5	L	P	Precuneus	19	AIP

10LD	FEAR								
Dipole No.	Magnitude E-06	T-x [mm]	T-y [mm]	T-z [mm]	Hem	Lobe	Gyrus	BA	ROI
625	4.201	50.8	-1.5	-27.5	R	T	Middle Temporal Gyrus	21	TEMP
878	3.194	-48.5	11.4	39	L	F	Middle Frontal Gyrus	8	DPLF
807	3.065	60.6	13.3	21.2	R	F	Inferior Frontal Gyrus	45	OBF
665	2.416	-8.5	-1.5	-27.5	L	Limbic	Uncus	28	LIMBIC
447	2.296	-69	-26	-8	L	T	Middle temporal Gyrus	21	TEMP
7	1.784	11.4	-97.5	2.1	R	O	Cuneus	18	OCC
99	1.647	-28.5	-81.9	39.5	L	P	Precuneus	19	AIP
70	1.404	31	-81.9	39.5	R	P	Precuneus	19	AIP
1048	1.244	-28.5	55.3	7.1	L	F	Middle Frontal Gyrus	10	OBF
647	1.060	31	-8.5	64.4	R	F	Precentral Gyrus	6	DPLF

11SG	FEAR								
Dipole No.	Magnitude E-06	T-x [mm]	T-y [mm]	T-z [mm]	Hem	Lobe	Gyrus	BA	ROI
172	3.155	60.6	-55	-17.6	R	O	Fusiform Gyrus	37	FUSIF
20	3.025	40.9	-85.8	-11.7	R	O	Inferior Occipital Gyrus	18	OCC
625	2.699	50.8	-1.5	-27.5	R	T	Middle Temporal Gyrus	21	TEMP
1019	2.495	-38.5	46	-2.1	L	F	Inferior Frontal Gyrus	10	OBF
1053	2.422	2.1	64.7	8	R	F	Medial Frontal Gyrus	10	OBF
318	2.281	60.6	-41.5	42.9	R	P	Inferior Parietal Lobule	40	AIP
893	2.053	31	19.8	48.5	R	F	Superior Frontal Gyrus	8	DPLF
242	1.908	-59	-55	-17.6	L	T	Fusiform Gyrus	37	FUSIF
665	1.875	-8.5	-1.5	-27.5	L	Limbic	Uncus	28	LIMBIC
920	1.545	-28.5	19.8	48.5	L	F	Superior Frontal Gyrus	8	DPLF
54	1.253	-27.5	-88.3	2.7	L	O	Middle Occipital Gyrus	18	OCC

12AC	FEAR								
Dipole No.	Magnitude E-06	T-x [mm]	T-y [mm]	T-z [mm]	Hem	Lobe	Gyrus	BA	ROI
172	9.606	60.6	-55	-17.6	R	O	Fusiform Gyrus	37	FUSIF
715	8.301	50.8	8	-19.8	R	T	Superior Temporal Gyrus	38	TEMP
930	7.749	50.8	36	-2.9	R	F	Inferior Frontal Gyrus	47	OBF
595	6.603	-28.5	-15.8	63	L	F	Precentral Gyrus	6	DPLF
325	4.897	50.8	-43	52	R	P	Inferior Parietal Lobule	40	AIP
665	4.199	-8.5	-1.5	-27.5	L	Limbic	Uncus	28	LIMBIC
889	4.074	40.9	21.2	39.5	R	F	Precentral Gyrus	9	DPLF
1019	3.441	-38.5	46	-2.1	L	F	Inferior Frontal Gyrus	10	OBF

14LD	FEAR								
Dipole No.	Magnitude E-06	T-x [mm]	T-y [mm]	T-z [mm]	Hem	Lobe	Gyrus	BA	ROI
2	2.942	21.2	-97	-13	R	O	Lingual Gyrus	18	OCC
949	2.636	11.4	29.7	58.8	R	F	Superior Frontal Gyrus	6	DPLF
803	1.703	-59	4.3	12	L	F	Precentral Gyrus	6	DPLF
106	1.531	-48.5	-76.2	-11.7	L	T	Fusiform Gyrus	19	FUSIF
94	1.257	-18.5	-81.9	39.5	L	P	Precuneus	19	AIP

15CC	FEAR								
Dipole No.	Magnitude E-06	T-x [mm]	T-y [mm]	T-z [mm]	Hem	Lobe	Gyrus	BA	ROI
448	3.568	71.2	-17.6	-7.1	R	T	Middle temporal Gyrus	21	TEMP
241	3.322	-48.5	-62	40.9	L	P	Inferior Parietal Lobule	39	AIP
1026	3.223	31	55.3	7.1	R	F	Middle Frontal Gyrus	10	OBF
242	3.181	-59	-55	-17.6	L	T	Fusiform Gyrus	37	FUSIF
15	2.681	-8	-99.4	11.4	L	O	Cuneus	18	OCC
793	2.659	-48.5	8	-19.8	L	T	Superior Temporal Gyrus	38	TEMP
916	2.484	-18.5	19.8	58	L	F	Superior Frontal Gyrus	6	DPLF
979	2.479	-48.5	34.9	4.9	L	F	Inferior Frontal Gyrus	45	OBF
204	2.466	11.4	-63.8	58.8	R	P	Superior Parietal Lobule	7	AIP
556	1.806	31	-15.8	63	R	F	Precentral Gyrus	6	DPLF
1	1.792	31	-97.5	-5.7	R	O	Inferior Occipital Gyrus	18	OCC
483	0.845	2	-19.8	27	R	Limbic	Cingulate Gyrus	23	LIMBIC

16MN	FEAR								
**Dipole No.**	**Magnitude E-06**	**T-x [mm]**	**T-y [mm]**	**T-z [mm]**	**Hem**	**Lobe**	**Gyrus**	**BA**	**ROI**
172	6.889	60.6	-55	-17.6	R	O	Fusiform Gyrus	37	FUSIF
310	6.192	71.2	-37	-1.5	R	T	Middle Temporal Gyrus	21	TEMP
977	5.311	-38.5	32	32	L	F	Middle Frontal Gyrus	9	DPLF
949	4.583	11.4	29.7	58.8	R	F	Superior Frontal Gyrus	6	DPLF
106	4.558	-48.5	-76.2	-11.7	L	T	Fusiform Gyrus	19	FUSIF
1025	3.931	31	56	-2.1	R	F	Superior Frontal Gyrus	10	OBF
30	3.300	21.2	-91.3	29.7	R	O	Cuneus	19	OCC
502	3.137	-38.5	-15	-29.6	L	T	Inferior Temporal Gyrus	20	TEMP
56	3.087	-28.5	-90.3	21.2	L	O	Middle Occipital Gyrus	19	OCC
1041	2.981	-8.5	56.8	-8.5	L	F	Superior Frontal Gyrus	10	OBF
198	2.534	21.2	-63.8	58.8	R	P	Superior Parietal Lobule	7	AIP
617	1.710	-59	-13.6	36.6	L	P	Supramarginal Gyrus	40	AIP

18MC	FEAR								
**Dipole No.**	**Magnitude E-06**	**T-x [mm]**	**T-y [mm]**	**T-z [mm]**	**Hem**	**Lobe**	**Gyrus**	**BA**	**ROI**
979	2.741	-48.5	34.9	4.9	L	F	Inferior Frontal Gyrus	45	OBF
702	2.314	-59	-1.5	-21	L	T	Middle temporal Gyrus	21	TEMP
530	2.057	60.6	-11.7	10.4	R	T	Transverse Temporal Gyrus	43	TEMP
211	1.412	2.1	-63.8	58.8	R	P	Precuneus	7	AIP
297	1.047	-39	-51	33	L	P	Inferior Parietal Lobule	40	AIP
22	1.040	40.9	-88.3	2.7	R	O	Middle Occipital Gyrus	18	OCC

19MR	FEAR								
**Dipole No.**	**Magnitude E-06**	**T-x [mm]**	**T-y [mm]**	**T-z [mm]**	**Hem**	**Lobe**	**Gyrus**	**BA**	**ROI**
1048	6.352	-28.5	55.3	7.1	L	F	Middle Frontal Gyrus	10	OBF
1024	3.569	40.9	55.3	7.1	R	F	Middle Frontal Gyrus	10	OBF
2	2.304	21.2	-97	-13	R	O	Lingual Gyrus	18	OCC
647	1.840	31	-8.5	64.4	R	F	Precentral Gyrus	6	DPLF
172	1.763	60.6	-55	-17.6	R	O	Fusiform Gyrus	37	FUSIF
171	1.687	-48.5	-71	31	L	P	Angular Gyrus	39	TEMP
400	1.494	21.2	-23.6	-15	R	Limbic	Parahippocampal Gyrus	35	LIMBIC
310	1.227	71.2	-37	-1.5	R	T	Middle Temporal Gyrus	21	TEMP

21GC	FEAR								
Dipole No.	Magnitude E-06	T-x [mm]	T-y [mm]	T-z [mm]	Hem	Lobe	Gyrus	BA	ROI
556	4.132	31	-15.8	63	R	F	Precentral Gyrus	6	DPLF
51	2.271	-18.5	-91.3	29.7	L	O	Cuneus	19	OCC
233	2.230	-38.5	-63	49.5	L	P	Superior Parietal Lobule	7	AIP
7	1.810	11.4	-97.5	2.1	R	O	Cuneus	18	OCC
172	1.708	60.6	-55	-17.6	R	O	Fusiform Gyrus	37	FUSIF
204	1.608	11.4	-63.8	58.8	R	P	Superior Parietal Lobule	7	AIP
242	1.536	-59	-55	-17.6	L	T	Fusiform Gyrus	37	FUSIF
678	1.535	-28.5	-1.5	-27.5	L	Limbic	Uncus	36	LIMBIC
793	1.520	-48.5	8	-19.8	L	T	Superior Temporal Gyrus	38	TEMP
526	1.450	60.6	-8.5	-21.7	R	T	Inferior Temporal Gyrus	20	TEMP
1056	1.288	-8.5	64.4	16.8	L	F	Superior Frontal Gyrus	10	OBF
950	1.147	2.1	38	-17.6	R	F	Medial Frontal Gyrus	11	OBF
805	1.090	-59	2	29.7	L	F	Precentral Gyrus	6	DPLF

23FN	FEAR								
Dipole No.	Magnitude E-06	T-x [mm]	T-y [mm]	T-z [mm]	Hem	Lobe	Gyrus	BA	ROI
233	4.978	-38.5	-63	49.5	L	P	Superior Parietal Lobule	7	AIP
1	2.918	31	-97.5	-5.7	R	O	Inferior Occipital Gyrus	18	OCC
335	2.536	21.2	-43.6	60.6	R	P	Superior Parietal Lobule	7	AIP
982	2.429	50.8	44.6	5.9	R	F	Middle Frontal Gyrus	46	DPLF
319	2.259	50.8	-33.7	-23.6	R	T	Fusiform Gyrus	20	FUSIF
625	2.257	50.8	-1.5	-27.5	R	T	Middle Temporal Gyrus	21	TEMP
807	2.038	60.6	13.3	21.2	R	F	Inferior Frontal Gyrus	45	OBF
703	1.589	-59	-2.1	-13	L	T	Middle temporal Gyrus	21	TEMP
361	1.467	-48.5	-33.7	-23.6	L	T	Fusiform Gyrus	20	FUSIF
665	1.240	-8.5	-1.5	-27.5	L	Limbic	Uncus	28	LIMBIC
974	1.206	-28.5	31	40.9	L	F	Middle Frontal Gyrus	8	DPLF

25DE	FEAR								
Dipole No.	Magnitude E-06	T-x [mm]	T-y [mm]	T-z [mm]	Hem	Lobe	Gyrus	BA	ROI
19	5.212	-28.5	-97.5	-5.7	L	O	Lingual Gyrus	18	OCC
21	2.633	40.9	-87	-4.8	R	O	Inferior Occipital Gyrus	18	OCC
582	1.730	-18.5	-8	-28.5	L	Limbic	Uncus	36	LIMBIC
694	1.602	-48.5	-1.5	-27.5	L	T	Inferior Temporal Gyrus	20	TEMP
740	1.567	21.2	8.8	-27.5	R	Limbic	Uncus	38	LIMBIC
840	1.553	2.1	18	-18.5	R	F	Rectal Gyrus	11	OBF
457	1.497	50.8	-15.8	-21.7	R	T	Fusiform Gyrus	20	FUSIF
1050	1.454	-28.5	53	25	L	F	Superior Frontal Gyrus	10	OBF
647	1.361	31	-8.5	64.4	R	F	Precentral Gyrus	6	DPLF
863	1.228	-28.5	10	56.8	L	F	Middle Frontal Gyrus	6	DPLF

26MP	FEAR								
Dipole No.	Magnitude E-06	T-x [mm]	T-y [mm]	T-z [mm]	Hem	Lobe	Gyrus	BA	ROI
27	2.625	31	-90.3	21.2	R	O	Middle Occipital Gyrus	19	OCC
19	1.788	-28.5	-97.5	-5.7	L	O	Lingual Gyrus	18	OCC
595	1.653	-28.5	-15.8	63	L	F	Precentral Gyrus	6	DPLF
982	1.409	50.8	44.6	5.9	R	F	Middle Frontal Gyrus	46	DPLF
885	1.367	40.9	27	-11.3	R	F	Inferior Frontal Gyrus	47	OBF
361	1.301	-48.5	-33.7	-23.6	L	T	Fusiform Gyrus	20	FUSIF
497	1.200	-28.5	-15	-29.6	L	Limbic	Uncus	20	LIMBIC
219	1.027	-8.5	-63.8	58.8	L	P	Superior Parietal Lobule	7	AIP
389	0.824	50.8	-32	53	R	P	Inferior Parietal Lobule	40	AIP
1047	0.787	-28.5	56	-2.1	L	F	Superior Frontal Gyrus	10	OBF

28FR	FEAR								
Dipole No.	Magnitude E-06	T-x [mm]	T-y [mm]	T-z [mm]	Hem	Lobe	Gyrus	BA	ROI
20	4.436	40.9	-85.8	-11.7	R	O	Inferior Occipital Gyrus	18	OCC
172	4.252	60.6	-55	-17.6	R	O	Fusiform Gyrus	37	FUSIF
595	3.463	-28.5	-15.8	63	L	F	Precentral Gyrus	6	DPLF
107	2.709	-48.5	-76.2	-4	L	O	Middle Occipital Gyrus	19	OCC
733	2.704	31	8.8	-27.5	R	T	Superior Temporal Gyrus	38	TEMP
211	2.670	2.1	-63.8	58.8	R	P	Precuneus	7	AIP
665	2.030	-8.5	-1.5	-27.5	L	Limbic	Uncus	28	LIMBIC
1007	2.009	2.1	40.9	50.8	R	F	Superior Frontal Gyrus	8	DPLF

29EM	FEAR								
Dipole No.	Magnitude E-06	T-x [mm]	T-y [mm]	T-z [mm]	Hem	Lobe	Gyrus	BA	ROI
1046	5.825	-18.5	52	33.9	L	F	Superior Frontal Gyrus	9	DPLF
1	4.687	31	-97.5	-5.7	R	O	Inferior Occipital Gyrus	18	OCC
828	4.226	31	10	56.8	R	F	Middle Frontal Gyrus	6	DPLF
242	3.175	-59	-55	-17.6	L	T	Fusiform Gyrus	37	FUSIF
49.5	2.811	-18.5	-90.3	21.2	L	O	Cuneus	18	OCC
610	2.622	-59	-8.5	-21.7	L	T	Inferior Temporal Gyrus	20	TEMP
582	2.521	-18.5	-8	-28.5	L	Limbic	Uncus	36	LIMBIC
625	2.296	50.8	-1.5	-27.5	R	T	Middle Temporal Gyrus	21	TEMP
471	2.204	31	-15	-29.6	R	Limbic	Uncus	20	LIMBIC
807	2.130	60.6	13.3	21.2	R	F	Inferior Frontal Gyrus	45	OBF

30CG	FEAR								
Dipole No.	Magnitude E-06	T-x [mm]	T-y [mm]	T-z [mm]	Hem	Lobe	Gyrus	BA	ROI
64	3.902	40.9	-80	22	R	T	Middle Temporal Gyrus	19	TEMP
47	3.119	-8.5	-91.3	29.7	L	O	Cuneus	19	OCC
714	2.721	60.6	2.1	29.7	R	F	Precentral Gyrus	6	DPLF
1051	2.603	11.4	64.7	8	R	F	Superior Frontal Gyrus	10	OBF
702	2.465	-59	-1.5	-21	L	T	Middle temporal Gyrus	21	TEMP
242	2.229	-59	-55	-17.6	L	T	Fusiform Gyrus	37	FUSIF
225	1.470	-18.5	-63.8	58.8	L	P	Superior Parietal Lobule	7	AIP

31AC	FEAR								
Dipole No.	Magnitude E-06	T-x [mm]	T-y [mm]	T-z [mm]	Hem	Lobe	Gyrus	BA	ROI
169	8.390	-48.5	-69	13.6	L	T	Middle Temporal Gyrus	39	TEMP
57	7.260	-38.5	-85.8	-11.7	L	O	Inferior Occipital Gyrus	18	OCC
2	4.091	21.2	-97	-13	R	O	Lingual Gyrus	18	OCC
175	3.323	60.6	-58	5.9	R	T	Middle Temporal Gyrus	21	TEMP
556	2.599	31	-15.8	63	R	F	Precentral Gyrus	6	DPLF
617	2.397	-59	-13.6	36.6	L	P	Supramarginal Gyrus	40	AIP
1051	1.625	11.4	64.7	8	R	F	Superior Frontal Gyrus	10	OBF
665	1.402	-8.5	-1.5	-27.5	L	Limbic	Uncus	28	LIMBIC
1047	1.270	-28.5	56	-2	L	F	Superior Frontal Gyrus	10	OBF

Below are the individual swLORETA reconstructions for the emotional state "joy". CHEER= Cheerfulness, that is “joy”.

06SC	CHEER								
Dipole No.	Magnitude E-06	T-x [mm]	T-y [mm]	T-z [mm]	Hem	Lobe	Gyrus	BA	ROI
1024	10.150	40.9	55.3	7.1	R	F	Middle Frontal Gyrus	10	OBF
20	9.755	40.9	-85.8	-11.7	R	O	Inferior Occipital Gyrus	18	OCC
711	6.744	60.6	4.9	2.7	R	T	Superior Temporal Gyrus	22	TEMP
19	6.227	-28.5	-97.6	-5.7	L	O	Lingual Gyrus	18	OCC
246	5.259	-59	-59	15	L	T	Superior Temporal Gyrus	22	TEMP
979	4.565	-48.5	34.9	4.9	L	F	Inferior Frontal Gyrus	45	OBF
1018	4.280	-28.5	42.9	33	L	F	Middle Frontal Gyrus	9	DPLF
665	1.998	-8.5	-1.5	-27.5	L	Limbic	Uncus	28	LIMBIC

07AM	CHEER								
**Dipole No.**	**Magnitude E-06**	**T-x [mm]**	**T-y [mm]**	**T-z [mm]**	**Hem**	**Lobe**	**Gyrus**	**BA**	**ROI**
106	4.182	-48.5	-76.2	-11.7	L	T	Fusiform Gyrus	19	FUSIF
22	3.961	40.9	-88.3	2.7	R	O	Middle Occipital Gyrus	18	OCC
709	3.683	-59	-5.7	36.6	L	F	Precentral Gyrus	6	DPLF
185	3.352	50.8	-62	40.9	R	P	Inferior Parietal Lobule	39	AIP
806	2.485	60.6	13.6	13.3	R	F	Inferior Frontal Gyrus	44	OBF
828	2.248	31	10.2	56.8	R	F	Middle Frontal Gyrus	6	DPLF
836	1.688	11.4	12	29.7	R	Limbic	Cingulate Gyrus	24	LIMBIC
665	1.562	-8.5	-1.5	-27.5	L	Limbic	Uncus	28	LIMBIC

08MS	CHEER								
**Dipole No.**	**Magnitude E-06**	**T-x [mm]**	**T-y [mm]**	**T-z [mm]**	**Hem**	**Lobe**	**Gyrus**	**BA**	**ROI**
27	4.169	31	-90.3	21.2	R	O	Middle Occipital Gyrus	19	OCC
110	3.115	50.8	-67	-2.9	R	T	Inferior Temporal Gyrus	19	TEMP
713	2.055	60.6	2.7	21.2	R	F	Precentral Gyrus	6	DPLF
946	1.421	11.4	33.9	13.6	R	Limbic	Anterior Cingulate	32	LIMBIC
106	1.325	-48.5	-76.2	-11.7	L	T	Fusiform Gyrus	19	FUSIF
372	1.318	-59	-39.5	25	L	P	Inferior Parietal Lobule	40	AIP
863	1.134	-28.5	10.2	56.8	L	F	Middle Frontal Gyrus	6	DPLF
611	1.117	-59	-8.5	-13.6	L	T	Inferior Temporal Gyrus	21	TEMP
1048	1.099	-28.5	55.3	7.1	L	F	Middle Frontal Gyrus	10	OBF

09AF	CHEER								
**Dipole No.**	**Magnitude E-06**	**T-x [mm]**	**T-y [mm]**	**T-z [mm]**	**Hem**	**Lobe**	**Gyrus**	**BA**	**ROI**
1025	5.029	31	56	-2.1	R	F	Superior Frontal Gyrus	10	OBF
172	4.950	60.6	-55	-17.6	R	O	Fusiform Gyrus	37	FUSIF
303	4.887	-59	-48.8	-16.9	L	T	Inferior Temporal Gyrus	20	TEMP
1031	4.438	22.1	53	25	R	F	Superior Frontal Gyrus	9	DPLF
1021	3.992	-38.5	43.6	15	L	F	Middle Frontal Gyrus	10	OBF
318	3.876	60.6	-41.5	42.9	R	P	Inferior Parietal Lobule	40	AIP
871	3.554	-38.5	10.2	48	L	F	Middle Frontal Gyrus	6	DPLF
8	2.755	11.4	-99.4	11.4	R	O	Cuneus	18	OCC
241	2.164	-48.5	-62	40.9	L	P	Inferior Parietal Lobule	39	AIP
572	1.659	2.1.1	-13	27.7	R	Limbic	Cingulate Gyrus	23	LIMBIC

10LD	CHEER								
Dipole No.	Magnitude E-06	T-x [mm]	T-y [mm]	T-z [mm]	Hem	Lobe	Gyrus	BA	ROI
247	4.324	60.6	-48.8	-16.9	R	T	Inferior Temporal Gyrus	20	TEMP
457	3.367	50.8	-15.8	-21.7	R	T	Fusiform Gyrus	20	FUSIF
981	3.087	-48.5	33	23.1	L	F	Middle Frontal Gyrus	46	DPLF
361	3.046	-48.5	-33.7	-23.6	L	T	Fusiform Gyrus	20	FUSIF
15	2.926	-8	-99.4	11.4	L	O	Cuneus	18	OCC
582	2.916	-18.5	-8	-28.5	L	Limbic	Uncus	36	LIMBIC
502	2.899	-38.5	-15	-29.6	L	T	Inferior Temporal Gyrus	20	TEMP
884	2.704	50.8	22	31	R	F	Middle Frontal Gyrus	9	DPLF
70	2.077	31	-81.9	39.5	R	P	Precuneus	19	AIP
1025	1.901	31	56	-2.1	R	F	Superior Frontal Gyrus	10	OBF

11SG	CHEER								
Dipole No.	Magnitude E-06	T-x [mm]	T-y [mm]	T-z [mm]	Hem	Lobe	Gyrus	BA	ROI
20	3.083	40.9	-85.8	-11.7	R	O	Inferior Occipital Gyrus	18	OCC
893	3.055	31	19.8	48.5	R	F	Superior Frontal Gyrus	8	DPLF
242	2.764	-59	-55	-17.6	L	T	Fusiform Gyrus	37	FUSIF
109	2.600	50.8	-66.1	-11.3	R	T	Fusiform Gyrus	19	FUSIF
783	2.600	-38.5	8.8	-27.5	L	T	Superior Temporal Gyrus	38	TEMP
177	2.387	60.6	-59.5	23.1	R	T	Superior Temporal Gyrus	39	TEMP
12	2.038	-8	-96.5	-13	L	O	Lingual Gyrus	17	OCC
999	1.972	2.1	48	-16.9	R	F	Medial Frontal Gyrus	11	OBF
1055	1.931	-8.5	64.7	8	L	F	Superior Frontal Gyrus	10	OBF
211	1.636	2.1	-63.8	58.8	R	P	Precuneus	7	AIP
471	1.616	31	-15	-29.6	R	Limbic	Uncus	20	LIMBIC
164	1.271	-38.5	-72	39.5	L	P	Precuneus	19	AIP

12AC	CHEER								
Dipole No.	Magnitude E-06	T-x [mm]	T-y [mm]	T-z [mm]	Hem	Lobe	Gyrus	BA	ROI
172	7.740	60.6	-55	-17.6	R	O	Fusiform Gyrus	37	FUSIF
242	6.940	-59	-55	-17.6	L	T	Fusiform Gyrus	37	FUSIF
141	6.051	2.1	-73	48.5	R	P	Precuneus	7	AIP
625	5.310	50.8	-1.5	-27.5	R	T	Middle Temporal Gyrus	21	TEMP
586	4.592	-18.5	-15.8	63	L	F	Precentral Gyrus	6	DPLF
569	4.264	11.4	-15.8	63	R	F	Precentral Gyrus	6	DPLF
1024	4.189	40.9	55.3	7.1	R	F	Middle Frontal Gyrus	10	OBF
59	4.002	-38.5	-88.3	2.7	L	O	Middle Occipital Gyrus	18	OCC
4	3.292	21.2	-99.4	2.1	R	O	Cuneus	18	OCC
1056	1.937	-8.5	64.4	16.8	L	F	Superior Frontal Gyrus	10	OBF

14LD	CHEER								
Dipole No.	Magnitude E-06	T-x [mm]	T-y [mm]	T-z [mm]	Hem	Lobe	Gyrus	BA	ROI
617	3.694	-59	-13.6	36.6	L	P	Supramarginal Gyrus	40	AIP
1	3.310	31	-97.6	-5.7	R	O	Inferior Occipital Gyrus	18	OCC
920	2.561	-28.5	19.8	48.5	L	F	Superior Frontal Gyrus	8	DPLF
793	2.526	-48.5	8	-19.8	L	T	Superior Temporal Gyrus	38	TEMP
354	2.233	-27.5	-35	-15.8	L	T	Fusiform Gyrus	20	FUSIF
884	2.158	50.8	22	31	R	F	Middle Frontal Gyrus	9	DPLF
564	1.350	11.4	-8.5	-13.6	R	Limbic	Parahippocampal Gyrus	34	LIMBIC
325	1.141	50.8	-43	52	R	P	Inferior Parietal Lobule	40	AIP

15CC	CHEER								
Dipole No.	Magnitude E-06	T-x [mm]	T-y [mm]	T-z [mm]	Hem	Lobe	Gyrus	BA	ROI
94	4.009	-18.5	-81.9	39.5	L	P	Precuneus	19	AIP
108	3.845	-48.5	-77.5	4.3	L	O	Middle Occipital Gyrus	19	OCC
970	3.731	-18.5	31	49.5	L	F	Superior Frontal Gyrus	8	DPLF
711	3.588	60.6	4.9	2.7	R	T	Superior Temporal Gyrus	22	TEMP
11	3.454	2.1	-99.4	11.4	R	O	Cuneus	18	OCC
325	3.177	50.8	-43	52	R	P	Inferior Parietal Lobule	40	AIP
982	2.925	50.8	44.6	5.9	R	F	Middle Frontal Gyrus	46	DPLF
980	2.911	-48.5	33.9	13.6	L	F	Middle Frontal Gyrus	10	OBF
1053	2.797	2.1	64.7	8	R	F	Medial Frontal Gyrus	10	OBF
242	2.475	-59	-55	-17.6	L	T	Fusiform Gyrus	37	FUSIF
502	1.602	-38.5	-15	-29.6	L	T	Inferior Temporal Gyrus	20	TEMP

16MN	CHEER								
Dipole No.	Magnitude E-06	T-x [mm]	T-y [mm]	T-z [mm]	Hem	Lobe	Gyrus	BA	ROI
1022	13.450	-38.5	42.9	24.2	L	F	Middle Frontal Gyrus	10	OBF
250	10.770	60.6	-48.5	5.9	R	T	Middle Temporal Gyrus	21	TEMP
20	10.240	40.9	-85.8	-11.7	R	O	Inferior Occipital Gyrus	18	OCC
1032	8.743	21.2	52	33.9	R	F	Superior Frontal Gyrus	9	DPLF
881	6.752	50.8	26	-4	R	F	Inferior Frontal Gyrus	47	OBF
108	5.726	-48.5	-77.5	4.3	L	O	Middle Occipital Gyrus	19	OCC

18MC	CHEER								
Dipole No.	Magnitude E-06	T-x [mm]	T-y [mm]	T-z [mm]	Hem	Lobe	Gyrus	BA	ROI
449	4.396	60.6	-15.8	-21.7	R	T	Inferior Temporal Gyrus	20	TEMP
318	3.645	60.6	-41.5	42.9	R	P	Inferior Parietal Lobule	40	AIP
624	2.850	60.6	-5.7	36.6	R	F	Precentral Gyrus	6	DPLF
665	2.632	-8.5	-1.5	-27.5	L	Limbic	Uncus	28	LIMBIC
439	2.520	-58	-23.6	-15	L	T	Middle temporal Gyrus	21	TEMP
36	2.173	11.4	-91.3	29.7	R	O	Cuneus	19	OCC
108	2.029	-48.5	-77.5	4.3	L	O	Middle Occipital Gyrus	19	OCC
1047	1.882	-28.5	56	-2.1	L	F	Superior Frontal Gyrus	10	OBF
272	1.622	11.4	-49.5	24.2	R	Limbic	Cingulate Gyrus	31	LIMBIC

19MR	CHEER								
Dipole No.	Magnitude E-06	T-x [mm]	T-y [mm]	T-z [mm]	Hem	Lobe	Gyrus	BA	ROI
1051	3.970	11.4	64.7	8	R	F	Superior Frontal Gyrus	10	OBF
753	1.315	11.4	-1.5	64.7	R	F	Superior Frontal Gyrus	6	DPLF
373	0.916	-59	-40.6	33.9	L	P	Supramarginal Gyrus	40	AIP
172	0.858	60.6	-55	-17.6	R	O	Fusiform Gyrus	37	FUSIF
110	0.812	50.8	-67	-2.9	R	T	Inferior Temporal Gyrus	19	TEMP
57	0.708	-38.5	-85.8	-11.7	L	O	Inferior Occipital Gyrus	18	OCC

21GC	CHEER								
Dipole No.	Magnitude E-06	T-x [mm]	T-y [mm]	T-z [mm]	Hem	Lobe	Gyrus	BA	ROI
556	4.914	31	-15.8	63	R	F	Precentral Gyrus	6	DPLF
242	3.032	-59	-55	-17.6	L	T	Fusiform Gyrus	37	FUSIF
502	2.379	-38.5	-15	-29.6	L	T	Inferior Temporal Gyrus	20	TEMP
292	2.229	-18.5	-54	59.5	L	P	Superior Parietal Lobule	7	AIP
172	2.127	60.6	-55	-17.6	R	O	Fusiform Gyrus	37	FUSIF
15	2.044	-8	-99.4	11.4	L	O	Cuneus	18	OCC
9	2.032	0.5	-97	-5.7	R	O	Lingual Gyrus	18	OCC
709	1.993	-59	-5.7	36.6	L	F	Precentral Gyrus	6	DPLF
113	1.917	50.8	-70	23.1	R	T	Middle Temporal Gyrus	39	TEMP
557	1.763	21.2	-8	-28.5	R	Limbic	Uncus	36	LIMBIC

23FN	CHEER								
Dipole No.	Magnitude E-06	T-x [mm]	T-y [mm]	T-z [mm]	Hem	Lobe	Gyrus	BA	ROI
27	2.478	31	-90.3	21.2	R	O	Middle Occipital Gyrus	19	OCC
233	2.022	-38.5	-63	49.5	L	P	Superior Parietal Lobule	7	AIP
647	1.343	31	-8.5	64.4	R	F	Precentral Gyrus	6	DPLF
932	1.318	50.8	33.9	13.6	R	F	Middle Frontal Gyrus	10	OBF
319	1.219	50.8	-33.7	-23.6	R	T	Fusiform Gyrus	20	FUSIF
625	1.168	50.8	-1.5	-27.5	R	T	Middle Temporal Gyrus	21	TEMP
335	1.068	21.2	-43.6	60.6	R	P	Superior Parietal Lobule	7	AIP
57	1.015	-38.5	-85.8	-11.7	L	O	Inferior Occipital Gyrus	18	OCC
582	0.999	-18.5	-8	-28.5	L	Limbic	Uncus	36	LIMBIC
471	0.985	31	-15	-29.6	R	Limbic	Uncus	20	LIMBIC
354	0.928	-27.5	-35	-15.8	L	T	Fusiform Gyrus	20	FUSIF
702	0.904	-59	-1.5	-21	L	T	Middle temporal Gyrus	21	TEMP
1018	0.888	-28.5	42.9	33	L	F	Middle Frontal Gyrus	9	DPLF

25DE	CHEER								
Dipole No.	Magnitude E-06	T-x [mm]	T-y [mm]	T-z [mm]	Hem	Lobe	Gyrus	BA	ROI
625	3.734	50.8	-1.5	-27.5	R	T	Middle Temporal Gyrus	21	TEMP
105	3.562	-39	-81	31	L	O	Superior Occipital Gyrus	19	OCC
319	3.484	50.8	-33.7	-23.6	R	T	Fusiform Gyrus	20	FUSIF
1052	2.720	11.4	64.4	16.8	R	F	Superior Frontal Gyrus	10	OBF
1034	2.101	11.4	52	33.9	R	F	Superior Frontal Gyrus	9	DPLF
63	1.866	40.9	-79	13.3	R	O	Middle Occipital Gyrus	19	OCC
665	1.764	-8.5	-1.5	-27.5	L	Limbic	Uncus	28	LIMBIC
774	1.608	-18.5	-1.5	64.7	L	F	Superior Frontal Gyrus	6	DPLF
211	1.532	2.1	-63.8	58.8	R	P	Precuneus	7	AIP
1015	1.303	-28.5	46	-2	L	F	Middle Frontal Gyrus	10	OBF
483	0.717	2.1	-19.8	27	R	Limbic	Cingulate Gyrus	23	LIMBIC

26MP	CHEER								
Dipole No.	Magnitude E-06	T-x [mm]	T-y [mm]	T-z [mm]	Hem	Lobe	Gyrus	BA	ROI
64.4	3.241	40.9	-80	22	R	T	Middle Temporal Gyrus	19	TEMP
22	3.177	40.9	-88.3	2.7	R	O	Middle Occipital Gyrus	18	OCC
685	1.894	-28.5	-8.5	64.4	L	F	Superior Frontal Gyrus	6	DPLF
16	1.626	-18.5	-97	-13	L	O	Lingual Gyrus	18	OCC
1040	1.108	2.1	52	33.9	R	F	Medial Frontal Gyrus	9	DPLF
318	1.032	60.6	-41.5	42.9	R	P	Inferior Parietal Lobule	40	AIP
665	0.987	-8.5	-1.5	-27.5	L	Limbic	Uncus	28	LIMBIC
610	0.926	-59	-8.5	-21.7	L	T	Inferior Temporal Gyrus	20	TEMP

28FR	CHEER								
Dipole No.	Magnitude E-06	T-x [mm]	T-y [mm]	T-z [mm]	Hem	Lobe	Gyrus	BA	ROI
21.2	4.692	40.9	-87	-4.8	R	O	Inferior Occipital Gyrus	18	OCC
113	4.640	50.8	-70	23.1	R	T	Middle Temporal Gyrus	39	TEMP
595	4.603	-28.5	-15.8	63	L	F	Precentral Gyrus	6	DPLF
980	4.576	-48.5	33.9	13.6	L	F	Middle Frontal Gyrus	10	OBF
959	3.480	2.1	29.7	58.8	R	F	Superior Frontal Gyrus	6	DPLF
106	2.965	-48.5	-76.2	-11.7	L	T	Fusiform Gyrus	19	FUSIF
219	2.508	-8.5	-63.8	58.8	L	P	Superior Parietal Lobule	7	AIP
582	2.047	-18.5	-8	-28.5	L	Limbic	Uncus	36	LIMBIC

29EM	CHEER								
Dipole No.	Magnitude E-06	T-x [mm]	T-y [mm]	T-z [mm]	Hem	Lobe	Gyrus	BA	ROI
556	5.703	31	-15.8	63	R	F	Precentral Gyrus	6	DPLF
447	5.328	-69	-26	-8	L	T	Middle temporal Gyrus	21	TEMP
1046	5.184	-18.5	52	33.9	L	F	Superior Frontal Gyrus	9	DPLF
582	4.974	-18.5	-8	-28.5	L	Limbic	Uncus	36	LIMBIC
354	4.768	-27.5	-35	-15.8	L	T	Fusiform Gyrus	20	FUSIF
625	4.681	50.8	-1.5	-27.5	R	T	Middle Temporal Gyrus	21	TEMP
4	4.514	21.2	-99.4	2.1	R	O	Cuneus	18	OCC
172	4.498	60.6	-55	-17.6	R	O	Fusiform Gyrus	37	FUSIF
1051	2.843	11.4	64.7	8	R	F	Superior Frontal Gyrus	10	OBF
211	2.373	2.1	-63.8	58.8	R	P	Precuneus	7	AIP
409	1.696	2.1	-28.5	26	R	Limbic	Cingulate Gyrus	23	LIMBIC

30CG	CHEER								
Dipole No.	Magnitude E-06	T-x [mm]	T-y [mm]	T-z [mm]	Hem	Lobe	Gyrus	BA	ROI
49.5	3.761	-18.5	-90.3	21.2	L	O	Cuneus	18	OCC
65	3.737	40.9	-81	31	R	O	Superior Occipital Gyrus	19	OCC
1026	3.607	31	55.3	7.1	R	F	Middle Frontal Gyrus	10	OBF
1034	2.604	11.4	52	33.9	R	F	Superior Frontal Gyrus	9	DPLF
242	2.447	-59	-55	-17.6	L	T	Fusiform Gyrus	37	FUSIF
702	2.447	-59	-1.5	-21	L	T	Middle temporal Gyrus	21	TEMP
219	2.041	-8.5	-63.8	58.8	L	P	Superior Parietal Lobule	7	AIP
970	1.481	-18.5	31	49.5	L	F	Superior Frontal Gyrus	8	DPLF

31AC	CHEER								
Dipole No.	Magnitude E-06	T-x [mm]	T-y [mm]	T-z [mm]	Hem	Lobe	Gyrus	BA	ROI
172	3.719	60.6	-55	-17.6	R	O	Fusiform Gyrus	37	FUSIF
1019	3.379	-38.5	46	-2.1	L	F	Inferior Frontal Gyrus	10	OBF
22	3.372	40.9	-88.3	2.7	R	O	Middle Occipital Gyrus	18	OCC
518	3.312	-59	-16.9	-15	L	T	Middle temporal Gyrus	21	TEMP
106	3.265	-48.5	-76.2	-11.7	L	T	Fusiform Gyrus	19	FUSIF
746	3.215	21.2	-1.5	64.7	R	F	Superior Frontal Gyrus	6	DPLF
625	3.162	50.8	-1.5	-27.5	R	T	Middle Temporal Gyrus	21	TEMP
950	2.858	2.1	38	-17.6	R	F	Medial Frontal Gyrus	11	OBF
141	2.826	2.1	-73	48.5	R	P	Precuneus	7	AIP
366	1.786	-48.5	-43	52	L	P	Inferior Parietal Lobule	40	AIP
